# A Phase I, Open-Label Trial, Evaluating the Safety and Immunogenicity of Candidate Tuberculosis Vaccines AERAS-402 and MVA85A, Administered by Prime-Boost Regime in BCG-Vaccinated Healthy Adults

**DOI:** 10.1371/journal.pone.0141687

**Published:** 2015-11-03

**Authors:** Sharon Sheehan, Stephanie A. Harris, Iman Satti, David A. Hokey, Veerabadran Dheenadhayalan, Lisa Stockdale, Zita-Rose Manjaly Thomas, Alice Minhinnick, Morven Wilkie, Samantha Vermaak, Joel Meyer, Matthew K. O’Shea, Maria Grazia Pau, Isabella Versteege, Macaya Douoguih, Jenny Hendriks, Jerald Sadoff, Bernard Landry, Paul Moss, Helen McShane

**Affiliations:** 1 The Jenner Institute, University of Oxford, Oxford, OX3 7DQ, United Kingdom; 2 Aeras Global TB Vaccine Foundation, Rockville, MD, United States of America; 3 Janssen Infectious Diseases and Vaccines (formerly Crucell), Leiden, The Netherlands; 4 School of Cancer Sciences, University of Birmingham, Edgbaston, Birmingham, B15 2TT, United Kingdom; The George Washington University School of Medicine and Health Sciences, UNITED STATES

## Abstract

**Background:**

MVA85A and AERAS-402 are two clinically advanced viral vectored TB vaccine candidates expressing *Mycobacterium tuberculosis* antigens designed to boost BCG-induced immunity. Clinical trials with candidate malaria vaccines have demonstrated that adenoviral vector based priming immunisation, followed by MVA vector boost, induced high levels of immunity. We present the safety and immunogenicity results of the first clinical trial to evaluate this immunisation strategy in TB.

**Methods:**

In this phase 1, open-label trial, 40 healthy previously BCG-vaccinated participants were enrolled into three treatment groups and vaccinated with 1 or 2 doses of AERAS-402 followed by MVA85A; or 3 doses of AERAS-402.

**Results:**

Most related adverse events (AEs) were mild and there were no vaccine related serious AEs. Boosting AERAS-402 with MVA85A significantly increased Ag85A-specific T-cell responses from day of vaccination. Two priming doses of AERAS-402 followed by MVA85A boost, resulted in a significantly higher AUC post-peak Ag85A response compared to three doses of AERAS-402 and historical data with MVA85A vaccination alone. The frequency of CD8+ T-cells producing IFN-γ, TNF-α and IL-2 was highest in the group receiving two priming doses of AERAS-402 followed by MVA85A.

**Conclusions:**

Vaccination with AERAS-402 followed by MVA85A was safe and increased the durability of antigen specific T-cell responses and the frequency and polyfunctionality of CD8+ T-cells, which may be important in protection against TB. Further clinical trials with adenoviral prime-MVA85A boost regimens are merited to optimise vaccination intervals, dose and route of immunisation and to evaluate this strategy in the target population in TB high burden countries.

**Trial Registration:**

ClinicalTrials.gov NCT01683773.

## Introduction

Tuberculosis (TB) remains a major global public health burden, with an estimated 9.0 million incident cases and 1.5 million deaths in 2013 [1]. Bacillus Calmette–Guérin (BCG), the only licensed vaccine, prevents disseminated disease in childhood. However, the protection conferred against pulmonary disease is highly variable [2–4]. A more effective vaccination strategy is urgently needed [5]. One potential strategy is to boost BCG with a recombinant viral vector encoding *Mycobacterium tuberculosis (M*.*tb)* specific antigens, thus retaining the benefits of BCG against disseminated disease.

MVA85A and AERAS-402 are two clinically advanced TB vaccine candidates. Both have been shown to boost immunity induced by prior BCG vaccination. MVA85A comprises the recombinant replication-deficient Modified Vaccinia virus Ankara expressing the immunodominant *M*.*tb* antigen 85A, and induced potent Ag85A-specific CD4+ T-cell responses in BCG vaccinated adults [6]. AERAS-402 comprises a recombinant replication-deficient adenovirus, serotype 35 (Ad35), expressing a fusion protein of three *M*.*tb* antigens Ag85A, Ag85B, and TB10.4. In adults, AERAS-402 induced a potent antigen-specific CD8+ T-cell response together with a less dominant CD4+ T cell response [7]. For optimal protective immunity against *M*.*tb*, a balanced CD4+ and CD8+ cellular immune response may be necessary [8, 9]. Preclinical simian immunodeficiency virus studies in macaques and human clinical trials with candidate malaria vaccines, have demonstrated that an adenoviral vector prime followed by an MVA vector boost is highly immunogenic [10, 11]. The aim of this phase I study was to evaluate the safety and immunogenicity of an AERAS-402 prime-MVA85A boost regime in BCG-vaccinated adults and to improve overall insert specific immunogenicity using a prime-boost combination.

## Materials and Methods

### Study design

This was a phase 1 open-label, non-randomized clinical vaccine with three treatment groups. The trial was approved by the Medicines and Healthcare products Regulatory Agency (MHRA, EudraCT 2012-002007-18), the Berkshire B Research Ethics Committee (reference12/SC/0283) and University Hospitals Birmingham NHS Foundation Trust Research and Development department (reference RRK4493). All participants provided written informed consent and the trial was conducted according to the principles of the Declaration of Helsinki and Good Clinical Practice. The CONSORT trial checklist and clinical trial protocol are available as S1 Protocol and S1 CONSORT Checklist.

### Participants

Subjects were recruited from the Centre for Clinical Vaccinology and Tropical Medicine, University of Oxford and the National Institute for Health Research (NIHR)/Wellcome Trust Clinical Research Facility at Birmingham. Screening and follow up visits occurred at both sites; all enrolment/vaccination visits occurred at the Oxford site.

Participants were BCG-vaccinated, healthy adults aged 18 to 55 with no clinically significant abnormalities in baseline haematology or biochemistry, negative serological testing for HIV, hepatitis B and hepatitis C viruses and a body mass index between 18 and 33 (kg/m^2^). Previous AERAS-402 clinical vaccine trials have included a Body Mass Index of > 33 kg/m^2^ as an exclusion factor; this was maintained in this study for consistency to allow comparison with previous clinical vaccine trials. Latent *M*.*tb* infection was excluded at screening by a negative T-SPOT.*TB* test (Oxford Immunotec).

### Treatment groups

AERAS-402 (1x10^11^ viral particles) was administered intramuscularly and MVA85A (1x10^8^ plaque forming units) was administered intradermally in the deltoid region of the upper arm. Subjects in Group A received AERAS-402 at study day (D) 0 and D28 and MVA85A at D119. Subjects in Group B received AERAS-402 at D0 and MVA85A at D56. Subjects in Group C received AERAS-402 at D0, D28 and D119.

### Sample size

**Fig 1:**
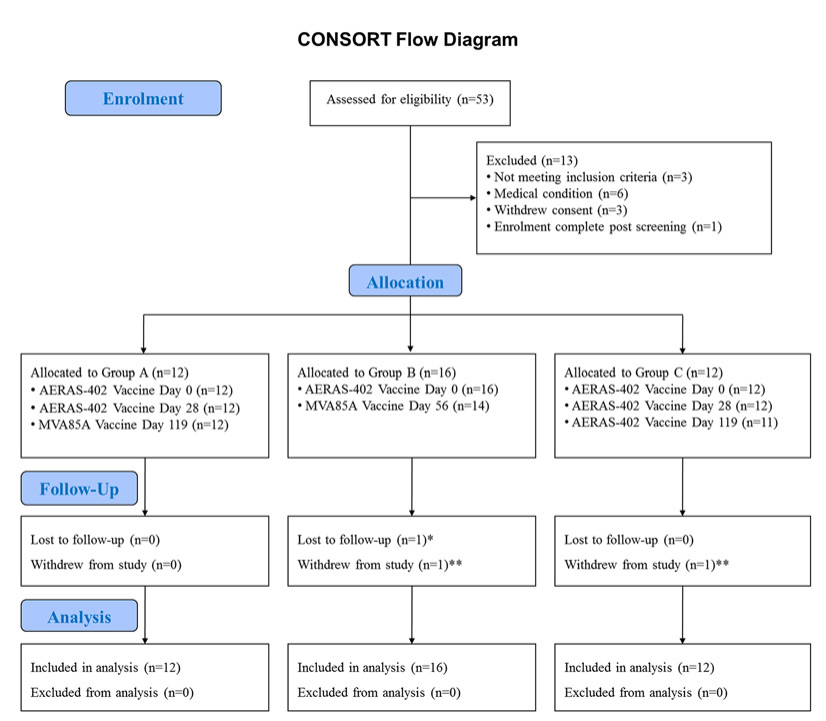
CONSORT flow diagram. CONSORT flow diagram showing subject recruitment and follow up. Subjects were allocated to Groups A and B in parallel and once both groups were complete, subjects were enrolled to Group C. *One subject was lost to follow up in Group B post day 14 follow up visit. All AEs post Day 0 vaccination had resolved prior to loss to follow up. **Two subjects withdrew from the study; one subject withdrew from Group B post first vaccination as did not wish to undergo any further venepunctures; one subject withdrew from Group C post second vaccination due to unexpected relocation. Both withdrawals were not considered related to vaccination and all AEs had resolved prior to withdrawal. The subject lost to follow up and the subject who withdrew from Group A was replaced; the subject who withdrew from Group C was not replaced as the study was nearing completion.

The initial plan was to enrol 15 subjects into each arm. However, due to challenges with enrolment the protocol was amended and 12 subjects were enrolled into Group A, 16 into Group B and 12 into Group C (Fig 1). Our previous experience suggests that this sample size is a feasible number to recruit, screen, enrol, and follow up in practical terms, whilst also allowing the determination of any substantial differences in the outcome measures between the three groups, including the frequency of AEs and SAEs and the size of the immune responses generated. The sample size has not been determined with the aim of achieving statistical significance. This sample size is appropriate for a proof-of-concept phase I safety trial.

Groups A and B were enrolled in parallel and once complete, subjects were enrolled into Group C. Subjects were not randomised to facilitate enrolment as the groups had different visit schedules.

### Study recruitment

Volunteers were enrolled within 90 days of screening visit. Volunteers in Groups A and C were followed up until day 203 (3 months post final vaccination visit at D119) and volunteers in Group B were followed up until day 140 (3 months post final vaccination visit at D56).

### Endpoints

The primary endpoint was safety as assessed by frequency and severity of vaccine-related local and systemic adverse events (AEs). Expected local AEs (pain, erythema, swelling, warmth, pruritus, scaling, axillary lymphadenopathy and axillary tenderness) and systemic AEs (documented fever >38°C, feverishness, malaise, arthralgia, headache, myalgia, nausea, vomiting, fatigue, diarrhoea, dysuria, conjunctivitis, sore throat and upper respiratory tract type symptoms (cough and rhinorrhoea)) were solicited from subjects using a diary card for 7 days post-vaccination and at each follow-up visit. Haematology and biochemistry analysis was conducted at screening, and at D28, 42, 119 and 126 for subjects in Groups A and C, and at D14, 56 and 63 for Group B.

The secondary endpoint was immunogenicity of vaccinations in all three groups. This was measured by the ex vivo interferon-gamma (IFN-γ) ELISpot assay performed on fresh peripheral blood mononuclear cells (PBMC) stimulated with pools of mycobacterial peptides, as well as flow cytometric estimation of T cell IFN-γ, TNF-α, and IL-2 production, as quantified by intracellular cytokine staining (ICS). Blood samples for immunological analysis were taken at each study visit for all subjects: Days 0 (first vaccination), 28,42,56,119,126,147 and 203 for volunteers in Groups A and C and Days 0,14,28,56,63,84 and 140 for volunteers in Group B.

### Protocol deviations

There were 21 protocol deviations during the trial period. 17 deviations were due to study visits which were out of specified time window as subjects could not attend on schedule due to unforeseen personal circumstances.

Safety bloods were omitted in error for one subject in Group A at D126; these bloods were within normal range when added at D147 visit.

Haematology safety bloods were collected in an incorrect vacutainer tube for one subject in Group C at D126 visit; these results were within normal range when repeated one week later.

Immunology bloods were omitted in error for one subject in Group A at D119 visit; these bloods were collected 4 days later, and within the window period for that study visit.

A lower dose of MVA85A (44 uL) was administered to one subject in Group B at D56 study visit as the subject had a vasovagal episode during vaccination.

These listed protocol deviations were not deemed to have an adverse impact on the safety of the subjects involved or on the scientific data collected during the trial period and therefore all data from all subjects was included in the analysis.

### Vaccines

MVA85A was constructed as previously described [6] and produced under Good Manufacturing Practice (GMP) conditions by IDT Biologika GmbH (Dessau-Rosslau, Germany). AERAS-402 was constructed as previously described [12] and produced under GMP conditions by Crucell Holland BV (Leiden, The Netherlands).

### Immunological assays

#### *Ex-vivo* IFN-γ ELISpot assay

Peripheral blood mononuclear cells (PBMC) were isolated from whole blood and interferon-gamma (IFN-γ) ELISpot assays were performed on freshly isolated PBMC from all subjects at 0, 28, 42, 56, 119, 126, 147 and 203 days post-vaccination (Groups A and C) and at 0, 14, 28, 56, 63, 84 and 140 days post-vaccination (Group B) as previously described [13] using the Human IFN-γ ELISpot (ALP) kit (Mabtech). Responses to the vaccine inserts were measured using the following antigens; Ag85A (single pool of 66 15mer peptides, overlapping by 10 amino acids), Ag85B (single pool of 69 15mer peptides, overlapping by 11 amino acids) and TB10.4 (single pool of 21 15mer peptides, overlapping by 11 amino acids). Responses to the Ad35 vector were measured using pools of peptides from the hexon protein of Ad35, including 2 pools that are specific to Ad35 and a pool that has some shared sequences with Ad26. Responses to these 3 pools were summed for analysis. Responses to MVA were measured using separate pools of CD4 (27 14-21mer peptides) and CD8 (36 9mer peptides) epitopes from vaccinia and MVA. All peptide pools were used at a final concentration of 2μg/mL/peptide. Staphylococcal enterotoxin B (SEB) (Sigma) (10μg/ml) was used as a positive control and unstimulated PBMC were used as a measure of background IFN-γ production. Results are reported as spot-forming cells (SFC) per million PBMC, calculated by subtracting the mean count of the unstimulated PBMC from the mean count of duplicate antigen wells and correcting for number of PBMC in the well.

#### 13-Color Intracellular cytokine staining assay

Intracellular cytokine staining (ICS) was performed as previously described [14]. PBMC were thawed and rested overnight. Cells (1 x 10^6^ cells/well) were stimulated in 96-well round bottom cell culture plates with Ag85A, Ag85B, and TB10.4 peptide pools (1 μg/peptide/mL), peptides were pre-diluted in R10 medium containing CD107a-Alexa488 (Biolegend, USA), GolgiStop and GolgiPlug (BD Biosciences, USA). Dimethyl sulfoxide (Sigma) was used as a negative control and SEB (0.5 μg/mL) as the positive control. Cells were stimulated for 6–7 hours at 37°C and 5% CO2. After incubation, the cells were washed and stained with viability dye (Invitrogen, USA) before staining with fluorochrome-conjugated antibodies to surface markers CD4 (eBioscience, USA), CD8 (Biolegend), CD14 and CD19 (BD), then permeabilized and stained for CD3 (Beckman Coulter, USA), IFN-γ, IL-2, CD154, TNF-α (all four from BD), IL-17A (Biolegend), and IL-22 (eBioscience). Cells were acquired on BD LSR flow cytometers (BD Biosciences). All sample analysis was performed with FlowJo software (TreeStar Inc., USA).

#### ELISA for anti-TB antibodies

Recombinant Ag85A, Ag85B, and TB10.4, purified in-house at Aeras, were coated onto micro-well plates. The serum samples were diluted 1:100 and incubated with immobilized antigen for capture of antigen specific antibodies present in the serum samples. The captured antibodies were then probed by the addition of biotinylated anti-IgG antibodies and detected using a colorimetric substrate solution. Once the optimum color intensity was reached, the color development was stopped using a stop solution. The micro-well plates were read using an ELISA reader with SoftMax® Pro 5.4.1 data acquisition and analysis software.

#### Adenovirus 35 neutralizing activity

The assay for Adenovirus 35 neutralizing activity was performed as previously described [15]. Briefly, control and sample sera were thawed, centrifuged, and then heat inactivated for 1 hour at 57°C. Samples were serially diluted in culture medium (DMEM/10%FBS/1% Pen Strep) in 96-well plates. Then 50 μL of Ad35.Luc (recombinant adenovirus 35 expressing a luciferase reporter construct) solution (1 x 10^8^ vp/mL) was added to each well. After incubation for neutralization, A549 cells were added at 1 x 104 cells/well, with a final ratio of 500 vp recombinant adenovirus/cell. After incubation at 37°C/10% CO_2_ for 24 to 26 hours, medium was discarded, PBS was added, and plates frozen at –35°C for a minimum of 12 hours. Plates were allowed to thaw at room temperature and Luciferase Steady-Lite Plus substrate was then added. After 15 minutes incubation, 50 μL of the lysate was transferred from each well into a B&W isoplate. Luminescence counts were recorded on a MicroBeta2. Data were imported into an Excel macro to calculate 90% inhibition titers. A response was defined as a titer >16, the lower limit of quantification for the serum neutralizing activity assay.

### Statistical analysis

Safety data were summarised by frequency and severity of adverse events using descriptive statistics. ELISpot data was analysed using GraphPad Prism. The Mann Whitney U-test and Area Under the Curve (AUC) analysis were used to detect differences between groups. The Wilcoxon matched-pairs signed rank test was used to detect differences between time points within the same group.

## Results

The first volunteer was enrolled in October 2012 and the trial was completed in August 2014, within the expected 24 months trial duration.

Fifty three subjects were assessed for eligibility of which 40 were enrolled (See Fig 1 for CONSORT flow diagram including reasons for exclusion).

The demographics of the enrolled subjects were comparable between the three groups (Table 1).

**Table 1:** Demographics of enrolled subjects.

		Vaccine Group		
		Group A (n = 12)	Group B (n = 16)	Group C (n = 12)
**Characteristic**	Male, n (%)	7 (58%)	7 (44%)	7 (58%)
	Median age in years (range)	23 (19–51)	26 (19–54)	27 (20–54)
	Median time interval since BCG in years (range)	9 (2–30)	19 (1–41)	12 (0–46)
	BMI (kg/m^2^) (range)	25 (20–32)	25 (18–30)	24 (19–31)
**Continent of birth**	Europe	12	14	10
	Asia	0	1	2
	Australia	0	1	0

Abbreviations: BCG, Bacillus Calmette–Guérin; BMI, Body mass index.

### Safety evaluation

There were 908 adverse events (AEs) of which 708 (78%) were considered related to vaccination. Most AEs were mild or moderate (Table 2).

**Table 2:** Summary of Adverse Events.

		Group A			Group B		Group C			Total AEs
**Severity**	Mild	285			208		224			717 (79%)
** **	Moderate	70			55		43			168 (19%)
** **	Severe	7			2		14			23 (2%)
**Total AEs**		**362**			**265**		**281**			**908 (100%)**
**AEs related to vaccine**	** **	**278 (77%)**			**208 (79%)**		**222 (79%)**			**708 (78%)**
**AEs related to vaccine**		**AERAS-402**	**AERAS-402**	**MVA85A**	**AERAS-402**	**MVA85A**	**AERAS-402**	**AERAS-402**	**AERAS-402**	
** **		Day 0 (n = 12)	Day 28 (n = 12)	Day 119 (n = 12)	Day 0 (n = 16)	Day 56 (n = 14)	Day 0 (n = 12)	Day 28 (n = 12)	Day 119 (n = 11)	
**Local AEs**	Mild	26	23	59	23	73	27	22	19	272 (87%)
** **	Moderate	5	5	4	11	4	1	5	0	35 (11%)
** **	Severe	1	2	0	0	0	2	0	0	5 (2%)
** **	**Total**	**32**	**30**	**63**	**34**	**77**	**30**	**27**	**19**	**312 (100%)**
**Systemic AEs**	Mild	32	47	24	46	22	39	44	32	286 (72%)
** **	Moderate	25	18	7	22	7	15	9	0	103 (26%)
** **	Severe	0	0	0	0	0	3	1	3	7 (2%)
** **	**Total**	**57**	**65**	**31**	**68**	**29**	**57**	**54**	**35**	**396 (100%)**

Abbreviations: AEs, adverse events.

One unrelated Serious Adverse Event occurred in a subject 80 days post final vaccination in Group A. This subject was diagnosed with presumptive Dengue fever while travelling in an endemic region, hospitalised for 2 days for supportive treatment and made a full recovery.

23 severe AEs were reported of which 12 were considered related to vaccination and occurred post AERAS-402 vaccination. Four subjects recorded transient severe pain (2 in Group A and 2 in Group C) and one subject recorded transient severe swelling at the vaccination site after AERAS-402.

One subject reported feverishness and malaise on D1 post first AERAS-402 vaccination requiring one day absence from work; this subject also recorded a fever (39.3°C) on the day of vaccination which returned to 37.3°C within 6 hours. This same subject reported nausea and abdominal pain post the third AERAS-402 vaccination requiring one day absence from work, with resolution of symptoms by D2 post vaccination. Another subject reported severe headache on the evening of third AERAS-402 vaccination which resolved within 24 hours. These AEs was assigned possible causality due to temporal association with vaccination.

21 laboratory abnormalities were detected of which 13 (11 mild, 1 moderate and 1 severe) were considered related to AERAS-402 vaccination. There were no laboratory AEs related to MVA85A vaccination. One subject in Group C had a moderate increase in alanine transaminase and a severe increase in aspartate aminotransferase post second AERAS-402 vaccination. Both returned to baseline prior to third vaccination and were considered possibly related to vaccination due to a temporal association with vaccination. All related laboratory AEs were transient, asymptomatic and returned to baseline by the end of the trial. All related AEs (including laboratory AEs) are listed in Table 3.

**Table 3:** The numbers of subjects within each group reporting each related adverse event.

		Group A			Group B		Group C		
Adverse events	Vaccine	AERAS-402	AERAS-402	MVA85A	AERAS-402	MVA85A	AERAS-402	AERAS-402	AERAS-402
		Day 0 (n = 12)	Day 28 (n = 12)	Day 119 (n = 12)	Day 0 (n = 16)	Day 56 (n = 14)	Day 0 (n = 12)	Day 28 (n = 12)	Day 119 (n = 11)
**Solicited local AEs**	Axillary lymphadenopathy	2(17%)	0(0%)	3(25%)	0(0%)	3(21%)	0(0%)	0(0%)	0(0%)
	Axillary tenderness	0(0%)	1(8%)	2(17%)	0(0%)	1(7%)	1(8%)	1(8%)	0(0%)
	Local erythema	6(50%)	6(50%)	12(100%)	8(50%)	14(100%)	9(75%)	7(58%)	5(45%)
	Local pain	12(100%)	10(83%)	9(75%)	15(94%)	9(64%)	12(100%)	11(92%)	8(73%)
	Local pruritus	2(17%)	1(8%)	6(50%)	1(6%)	10(71%)	1(8%)	0(0%)	0(0%)
	Local scaling	0(0%)	0(0%)	10(83%)	0(0%)	13(93%)	0(0%)	0(0%)	0(0%)
	Local swelling	3(25%)	4(33%)	12(100%)	2(13%)	14(100%)	6(50%)	5(42%)	4(36%)
	Local warmth	6(50%)	5(42%)	8(67%)	8(50%)	10(71%)	1(8%)	3(25%)	1(9%)
**Unsolicited local AEs**	Injection site bruise	0(0%)	0(0%)	0(0%)	0(0%)	0(0%)	0(0%)	0(0%)	1(9%)
**Solicited systemic AEs**	Arthralgia	3(25%)	5(42%)	2(17%)	3(19%)	2(14%)	5(42%)	4(33%)	3(27%)
	Cough	1(8%)	3(25%)	0(0%)	1(6%)	1(7%)	0(0%)	1(8%)	0(0%)
	Diarrhoea	2(17%)	1(8%)	0(0%)	2(13%)	0(0%)	1(8%)	1(8%)	0(0%)
	Fatigue	8(67%)	9(75%)	6(50%)	8(50%)	5(36%)	10(83%)	8(67%)	6(55%)
	Documented fever	2(17%)	3(25%)	0(0%)	0(0%)	0(0%)	2(17%)	1(8%)	0(0%)
	Felt feverish	10(83%)	8(67%)	3(25%)	9(56%)	5(36%)	9(75%)	7(58%)	7(64%)
	Headache	9(75%)	8(67%)	5(42%)	10(63%)	8(57%)	10(83%)	8(67%)	6(55%)
	Malaise	7(58%)	6(50%)	5(42%)	8(50%)	3(21%)	8(67%)	8(67%)	7(64%)
	Myalgia	7(58%)	7(58%)	2(17%)	8(50%)	3(21%)	7(58%)	7(58%)	2(18%)
	Nausea	0(0%)	2(17%)	1(8%)	5(31%)	0(0%)	1(8%)	1(8%)	2(18%)
	Rhinorrhoea	2(17%)	5(42%)	4(33%)	2(13%)	1(7%)	1(8%)	1(8%)	0(0%)
	Sore eyes	1(8%)	2(17%)	1(8%)	3(19%)	0(0%)	0(0%)	0(0%)	0(0%)
	Sore throat	2(17%)	1(8%)	2(17%)	2(13%)	1(7%)	1(8%)	1(8%)	0(0%)
	Vomiting	1(8%)	0(0%)	0(0%)	0(0%)	0(0%)	0(0%)	0(0%)	1(9%)
**Unsolicited systemic AEs**	Abdominal pain	0(0%)	0(0%)	0(0%)	0(0%)	0(0%)	0(0%)	0(0%)	1(9%)
	Chills	0(0%)	1(8%)	0(0%)	2(13%)	0(0%)	0(0%)	0(0%)	0(0%)
	Insomnia	0(0%)	0(0%)	0(0%)	1(6%)	0(0%)	0(0%)	0(0%)	0(0%)
	Lightheadedness	0(0%)	0(0%)	0(0%)	1(6%)	0(0%)	0(0%)	0(0%)	0(0%)
	Loss of appetite	0(0%)	0(0%)	0(0%)	1(6%)	0(0%)	0(0%)	0(0%)	0(0%)
	Migraine	0(0%)	1(8%)	0(0%)	0(0%)	0(0%)	0(0%)	0(0%)	0(0%)
	Night sweats	0(0%)	0(0%)	0(0%)	1(6%)	0(0%)	0(0%)	0(0%)	0(0%)
	Rigors	0(0%)	1(8%)	0(0%)	0(0%)	0(0%)	0(0%)	0(0%)	0(0%)
**Laboratory AEs**	Elevated ALT	0(0%)	0(0%)	0(0%)	0(0%)	0(0%)	0(0%)	2(17%)	0(0%)
	Elevated AST	0(0%)	0(0%)	0(0%)	0(0%)	0(0%)	0(0%)	2(17%)	0(0%)
	Hyperbilirubinaemia	1(8%)	0(0%)	0(0%)	1(6%)	0(0%)	0(0%)	0(0%)	0(0%)
	Lymphopaenia	0(0%)	0(0%)	0(0%)	0(0%)	0(0%)	0(0%)	1(8%)	0(0%)
	Neutropaenia	1(8%)	2(17%)	0(0%)	0(0%)	0(0%)	2(17%)	1(8%)	0(0%)

Local adverse events that were reported twice by individual subjects (8 adverse events) in the seven day diary card period are included in the summary adverse event Table 2.

All related adverse events were reported during the seven day diary card period apart from one volunteer who reported axillary tenderness at day 20 post second AERAS-402 vaccination which was deemed possibly related to vaccination. Abbreviations: AEs, adverse events; ALT, alanine transaminase; AST, aspartate aminotransferase.

### Vaccine immunogenicity

#### Boosting AERAS-402 with MVA85A induces strong and durable antigen-specific T- cell responses

**Fig 2:**
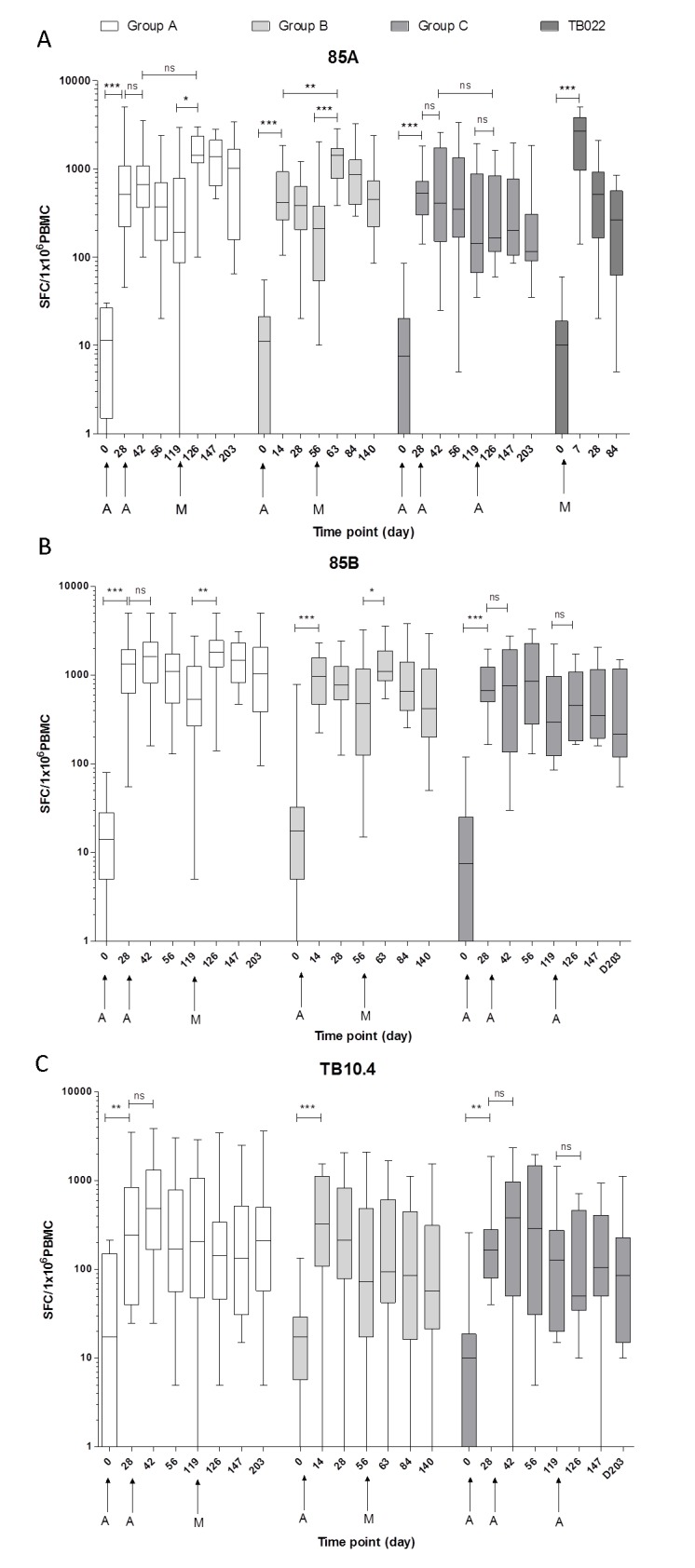
*Ex-vivo* IFN-γ ELISpot insert responses. Responses to Ag85A (A), Ag85B (B) and TB10.4 (C) in BCG-vaccinated, healthy adults for Group A (AERAS-402, AERAS-402, MVA85A), Group B (AERAS-402, MVA85A) and Group C (AERAS-402, AERAS-402, AERAS-402). Responses to Ag85A are also shown from TB022 (MVA85A). Box and whisker plots show median, inter-quartile range, minimum and maximum values. Stars denote significant changes in responses after vaccination (Wilcoxon matched pairs) * p = < 0.05, ** p = < 0.01, *** p = < 0.001, ns = not significant.

In all groups, ex-*vivo* IFN-γ ELISpot responses to Ag85A, Ag85B and TB10.4 increased significantly after first vaccination with AERAS-402 (Fig 2). A second AERAS-402 vaccination at D28 (Groups A and C) or a third AERAS-402 vaccination at D119 (Group C) did not boost these responses further. Boosting AERAS-402 with MVA85A significantly increased Ag85A responses in Group A and B, although the response did not significantly differ between these groups. The peak response post-MVA85A in Group A (D126) was not significantly higher than the peak response after two doses of AERAS-402 (D42). In Group B, the peak response post- MVA85A (D63) was significantly higher than the peak response after AERAS-402 (D14) (p = 0.0017). Responses to Ag85B significantly increased after MVA85A. MVA85A had no effect on responses to TB10.4 peptides. All three vaccination regimes induced responses that remained significantly above baseline at all time points except TB10.4 responses in Group A which returned to baseline at D147.

#### Two doses of AERAS-402 increases the magnitude of responses after MVA85A

Area under the curve (AUC) analysis was performed on Groups A, B, C and subjects from a previous trial, TB022, who received MVA85A alone [13]. Due to the different time points in the groups, the AUC analysis was performed on each subject’s responses to Ag85A, from 1 week post-MVA85A (or post third AERAS-402 for Group C) to 12 weeks post-MVA85A or third AERAS-402. Group A had a median AUC of 13,980 SFC/million PBMC, which was significantly higher than Group C (p = 0.0023) and the MVA85A alone group (p = 0.0464), with an AUC of 2074 and 7039 respectively. The median AUC for Group B was 6588 SFC/million PBMC and was not significantly different to Group A or to the MVA85A alone group, but was significantly higher than Group C (p = 0.0109).

#### Both AERAS-402 and MVA85A vaccination induce anti-vector responses

**Fig 3:**
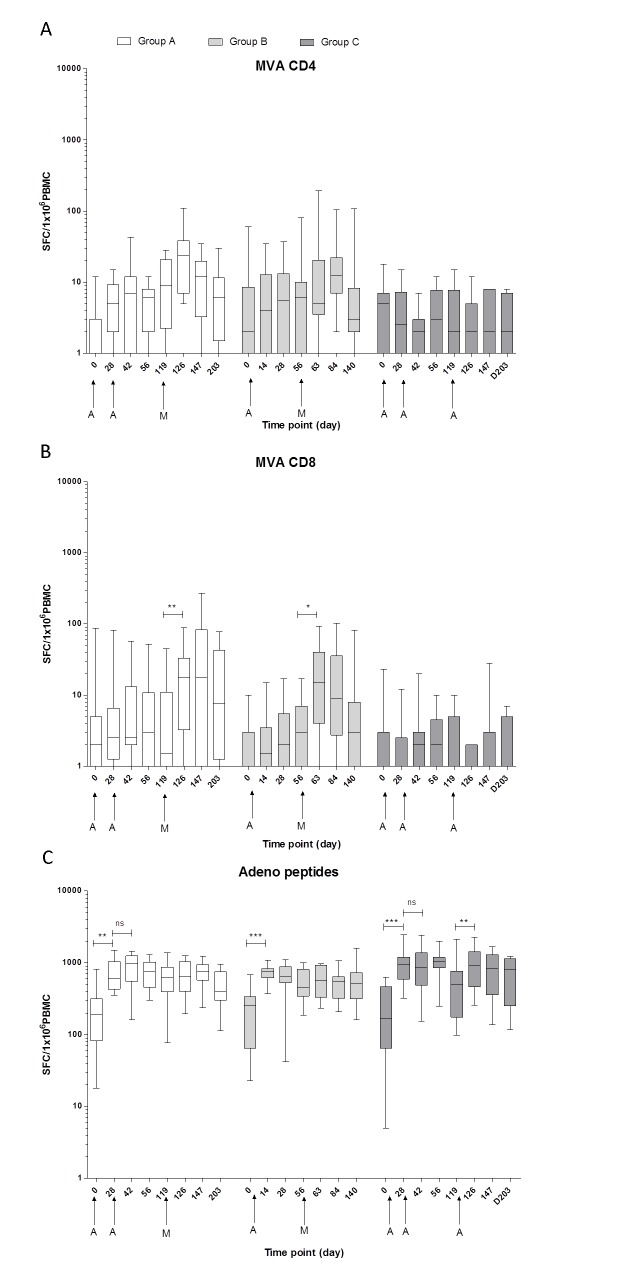
Ex-*vivo* IFN-γ ELISpot vector responses. Responses to MVA peptides for CD4+ (A), and CD8+ T-cell epitopes (B) and summed responses to 3 pools of adenovirus peptides (C) in BCG-vaccinated, healthy adults for Group A (AERAS-402, AERAS-402, MVA85A), Group B (AERAS-402, MVA85A) and Group C (AERAS-402, AERAS-402, AERAS-402). Box and whisker plots show median, inter-quartile range, minimum and maximum values. Stars denote significant changes in responses after vaccination (Wilcoxon matched pairs) * p = < 0.05, ** p = < 0.01, *** p = < 0.001, ns = not significant.

There was no difference between groups in their baseline responses to the adenovirus peptides. After first vaccination with AERAS-402, responses in all groups increased significantly (Fig 3. p = 0.001, 0.0001 and 0.0005 for Groups A, B and C respectively). There was no further increase in anti-vector responses after a second AERAS-402 vaccination, but after a third AERAS-402 vaccination (Group C), a significant increase was seen (p = 0.001). In Group A, responses to the adenovirus peptides remained above baseline until D147, whereas in Groups B and C, responses remained above baseline at all time points. There was a small, non-significant increase in responses to the CD4+ epitopes from MVA85A and a stronger, significant increase in responses to the CD8+ epitopes after vaccination with MVA85A. These CD8+ responses had all returned to baseline by the final time point.

#### Total intracellular cytokine response

**Fig 4:**
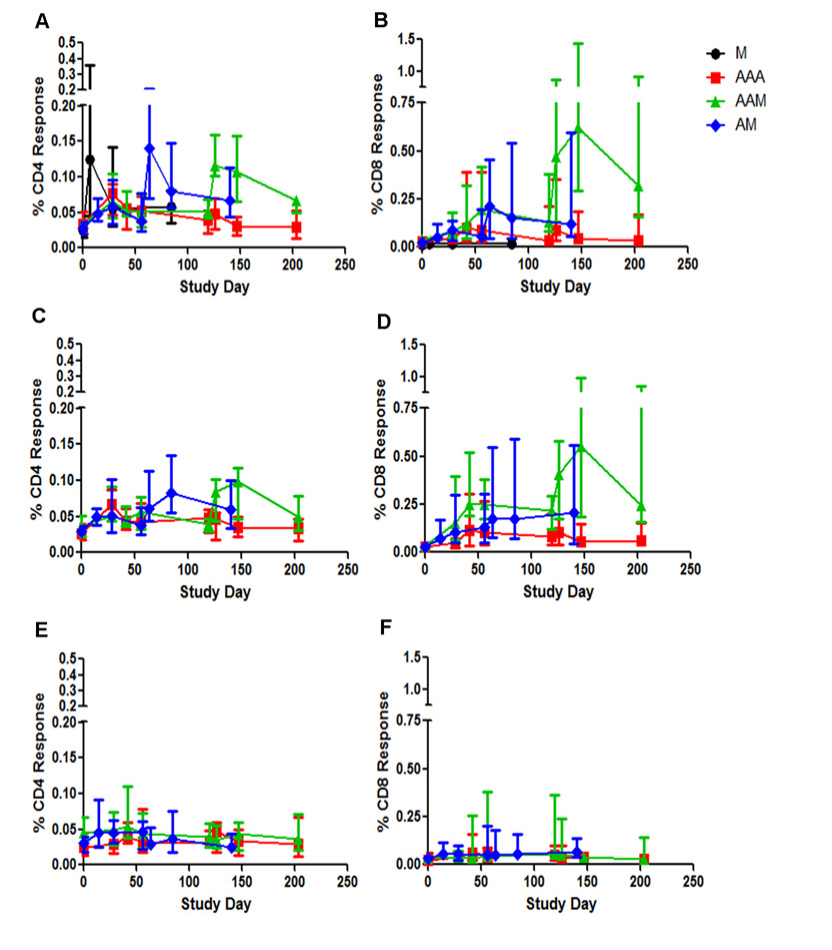
Total intracellular cytokine responses. Total intracellular cytokine responses are presented as percentages of CD4+ T-cells or CD8+ T-cells producing mycobacteria-specific IFN-γ, TNF-α and IL-2 cytokines. Percentage of CD4+ (A, C and E) and CD8+ (B, D and F) responses in peripheral blood mononuclear cells to stimulation with Ag85A (A and B), Ag85B (C and D) and TB10.4 (E and F) peptides in healthy, BCG-vaccinated adults from Group A (AAM, green line), Group B (AM, blue line), Group C (AAA, red line) and TB022 (M, black line). Lines show median responses in each group, whiskers show inter-quartile range.

AUC was done at week 1, 4 and 12 post MVA85A for Group A, B and MVA85A alone [13] and at the same time points after the third AERAS-402 in Group C. AUC analysis shows that Group A had significantly higher Ag85A-specific CD4+ and CD8+ T-cell responses than Group C (Fig 4A and 4B; p = 0.0018 and p = 0.0045 respectively). The same was true for Ag85B responses (Fig 4C and 4D; CD4+ T-cells p = 0.0036; CD8+ T-cells p = 0.0002). No differences were detected in the TB10.4 responses between Group A and C (Fig 4E and 4F).

No significant differences were detected between Group B and Group C in CD4+ T-cell or CD8+ T-cell responses to any antigens. Both the CD4+ and CD8+ T-cell response were significantly higher in Group A as compared to Group B in response to Ag85A (CD4 p = 0.0127; CD8 p = 0.0172) and Ag85B (CD4 p = 0.0173; CD8 p = 0.0233).

AUC analysis for the expression of the CD4+ T-cell activation marker, CD154, was significantly higher in Group A than in Group C in both Ag85A- and Ag85B-stimulated cells (p = 0.01 and p = 0.0068 respectively, data not shown).

After MVA85A vaccination, CD4+ and CD8+ T cell responses to Ag85A were higher in Group B when compared with MVA85A alone (Fig 4A and 4B; CD4 p = 0.0126; CD8 p<0.0001; AUC, Mann-Whitney).

#### Third vaccination with MVA85A but not AERAS-402 enhances intracellular mycobacteria specific CD4+ and CD8+ T-cell responses

Ag85A CD4+ T-cells cytokines were significantly higher in Group A than in Group C post third vaccination (Fig 4A; D126 p<0.0001; D147 p = 0.0023; D203 p = 0.0045). The Ag85B-specific CD4+ T-cell response was also higher in Group A than Group C (Fig 4C; D126 p = 0.0106; D147 p = 0.0057). CD8+ T-cell responses were also higher in Group A than Group C to Ag85A (Fig 4B; D126 p = 0.0372; D147 p = 0.0125; D203 p = 0.0106) and to Ag85B (Fig 4D; D119 p = 0.0196; D126 p = 0.0056; D147 p = 0.0057; D203 p = 0.0086). No significant differences were detected between groups in response to TB10.4 (Fig 4E and 4F). IL-17 and IL-22 were not detected.

#### Peak polyfunctional CD4+ and CD8+ T-cell responses

**Fig 5:**
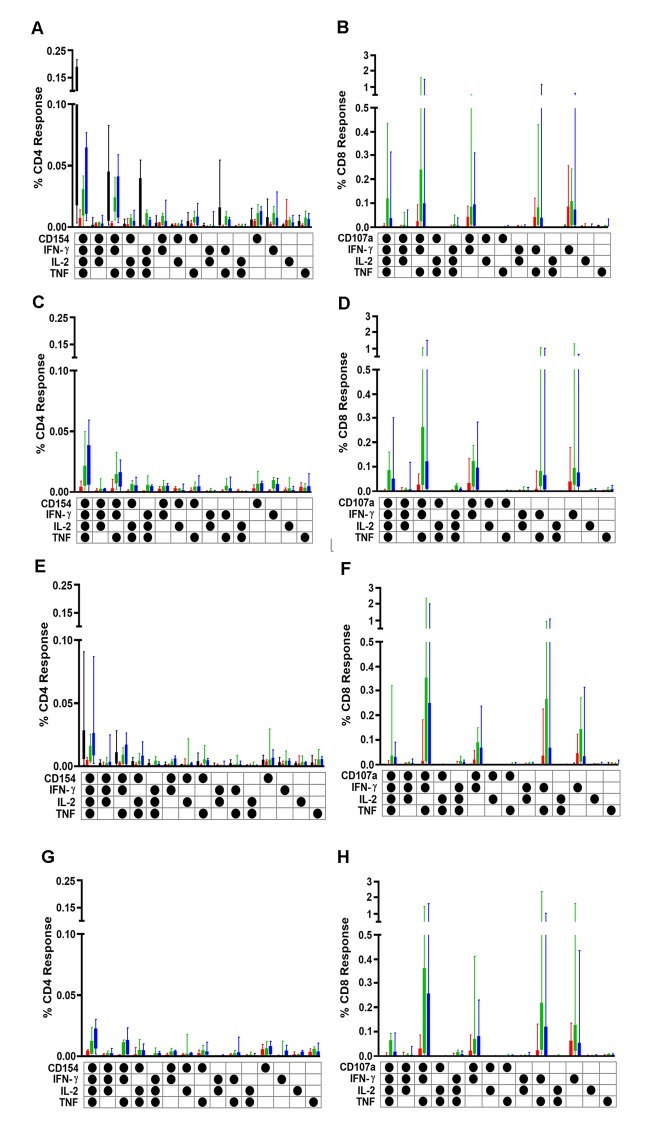
Polyfunctionality of peak and plateau intracellular cytokine responses. Polyfunctionality of CD4+ (A, C, E and G) and CD8+ (B, D, F and H) T-cells in response to stimulation with Ag85A and Ag85B peptides in healthy, BCG-vaccinated adults from Group A (AAM, green), Group B (AM, blue), Group C (AAA, red) and TB022 (M, black). Panels A-D show peak responses to Ag85A (A and B) and Ag85B (C and D), whereas panels E-H show plateau responses to Ag85A (E and F) and Ag85B (G and H). Plots show box and whisker with inter-quartile range and minimum and maximum values.

At peak time points, (D126 for Groups A and C, D63 for Group B and D7 for TB022), Group A had less Ag85A CD4+ polyfunctional positive T-cells than Group B. Groups A and B had more Ag85A-specific polyfunctional CD4+ T-cells than Group C. All three groups had less polyfunctional Ag85A-specific CD4+ T-cells than samples from TB022 (Fig 5A).

Polyfunctional CD8+ T-cells responses were only observed when MVA85A was preceded by AERAS-402 (Fig 5B). Groups A and B had comparable polyfunctional Ag85A CD8+ T-cell response, which were higher than Group C. No polyfunctional CD8+ T-cells were detected in the MVA85A alone group. Peak Ag85B polyfunctional CD4+ T-cells responses were lower in Group C than Groups A and B (Fig 5D).

#### Plateau polyfunctional CD4+ and CD8+ T-cell responses

At plateau, (D203 for Groups A and C, D140 for Group B and D84 for TB022), Group C had less polyfunctional Ag85A CD4+ T-cell response than all other groups (Fig 5E). No polyfunctional CD8+ T-cells were detected in the MVA85A alone group, Group C had less polyfunctional responses than Groups A and B (Fig 5F). Comparable plateau responses were detected in Ag85B CD4+ T-cells in Groups A and B, but Group C had less polyfunctional response (Fig 5G). Plateau polyfunctional Ag85B CD8+ T-cell responses were less in Group C compared to Groups A and B (Fig 5H).

#### Immunoglobulin G (IgG) responses

**Fig 6:**
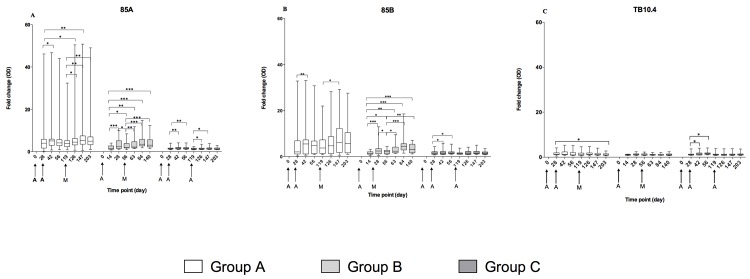
Serum antibody responses. Serum antibody responses to Ag85A (A), Ag85B (B) and TB10.4 (C) in the three study groups; Group A (AERAS-402, AERAS-402, MVA85A), Group B (AERAS-402, MVA85A) and Group C (AERAS-402, AERAS-402, AERAS-402). Antibody responses were measured in optical density (OD), data is presented as fold change responses calculated by dividing each time point’s antibody response by its corresponding day 0 response. Box and whisker plots show median, inter-quartile range, minimum and maximum values. Stars denote significant changes in responses after vaccination (Wilcoxon matched pairs) * p = < 0.05, ** p = < 0.01, *** p = < 0.001, ns = not significant.

Ag85A-specific IgG responses increased significantly after vaccination with AERAS-402 and MVA85A (Fig 6A). Group A responses peaked at D42 and were boosted by MVA85A vaccination to peak again on D147. A similar trend was observed in Group C, but to a lower magnitude than Group A, which had significantly higher Ag85A-IgG fold change at all time points (p < 0.05). Ag85A-specific IgG responses were significantly induced at all time points in Group B and were boosted with MVA85A vaccination. The peak Ag85A-IgG response four weeks after MVA85A vaccination was not significantly different between Groups A and B, however both of these groups had a significantly higher fold-change at this time point than the MVA85A alone group [13] and significantly higher than Group C (p < 0.05). At the last follow up, Ag85A-specific IgG was not significantly different between Group A (D203) and Group B (D140), while Group B had significantly higher fold change at this time point than Group C (D203) (p < 0.0001). A similar trend was observed in Ag85B-specific IgG responses (Fig 6B). The change in TB10.4-IgG response dropped significantly on D203 in Group A, but not Group C, which had a significant increase in TB10.4-IgG at D42 and D56. Differences in the magnitude of TB10.4-IgG were not significant between the three study groups.

#### Adenovirus 35 neutralising activity

AUC analyses for neutralising antibody responses show no significant differences between the three groups (data not shown).

## Discussion

We report the first TB clinical vaccine trial to evaluate an adenoviral prime—MVA boost regimen. The safety profile of AERAS-402 and MVA85A were comparable to data reported previously [7, 12, 13, 16, 17]. There were more expected local AEs post MVA85A vaccination than there were after AERAS-402 vaccination. These were mostly mild in severity and well tolerated. This is an expected finding as intradermal vaccinations induce more local reaction site reactions than intramuscular vaccinations, and this is consistent with our previous findings in an earlier trial comparing intramuscular versus intradermal delivery of MVA85A vaccine [13]. The frequency and severity of local AEs post MVA85A were consistent with previous trials [13, 16, 17]. As consistent with previous trials, there were no severe AEs related to MVA85A.

The vast majority of systemic AEs were mild or moderate in nature. There were 7 severe systemic AEs that were considered related to AERAS-402 vaccination, 4 of which were considered possibly related due to temporal association with vaccination. All of the vaccine related severe systemic AEs, apart from the transient rise in AST discussed below, resolved within 48 hours. The most objective measurement of systemic reactogenicity, pyrexia, was reported as severe on only one occasion throughout the study, a recorded fever of 39.3°C on the day of first AERAS-402 vaccination in a volunteer in Group C, which returned to normal within 6 hours. There was one severe laboratory AE, a transient elevation in AST post second AERAS-402 vaccination in Group C, which normalised prior to third vaccination. This AE was deemed possibly related to vaccination due to temporal association; the rise in AST may also have been attributable to excessive exercise prior to safety bloods measurement. There were no vaccination deferrals and there were no study withdrawals attributable to related AEs.

Ex-*vivo* IFN-γ ELISpot responses showed that while the first AERAS-402 vaccination significantly increased antigen specific T-cell responses to Ag85A, Ag85B and TB10.4, homologous boosting at D28 and D119 did not increase these responses further. Boosting AERAS-402 with MVA85A induced strong and durable antigen specific T-cell responses to antigen 85A, with two priming doses of AERAS-402 resulting in a significantly higher AUC post-peak MVA85A vaccination than MVA85A alone. After one dose of AERAS-402, the AUC post-MVA85A response was not significantly higher than after MVA85A alone. Anti-vector adenoviral T cell responses were increased after AERAS-402 vaccination and remained above baseline for the duration of the trial. Anti-MVA CD8+ T cell responses were induced after vaccination with MVA85A, but returned to baseline by the end of the trial. Polyfunctional CD4+ and CD8+ T cells responses were comparable when MVA85A was preceded by one (Group B) or two (Group A) AERAS-402 vaccinations.

Multiparameter flow cytometry showed a high frequency of cytokine producing CD8+ T-cells in Ag85A and Ag85B-stimulated PBMC after MVA85A boost in Groups A and B, with Group A showing a higher frequency post-boost. At peak response post-MVA85A, the frequency of polyfunctional CD4+ T-cells producing IFN-γ, TNF-α and IL-2, and expressing CD154, a marker of CD4+ T-cell activation, in response to stimulation with Ag85A, was highest in the MVA85A alone group, followed by Group B and then Group A. However, the frequency of CD8+ T-cells producing all 3 cytokines and upregulating the expression of CD107a, an indicator of CD8+ T-cell cytotoxicity, was highest in Group A, followed by Group B and barely detectable in the MVA85A alone group. The same trend is true for plateau responses, but with reduced frequencies.

Ag85A-specific IgG responses peaked four weeks after vaccination with MVA85A, but did not significantly differ between the groups receiving either one or two doses of AERAS-402 prime. However, responses were higher in both of the Adenovirus prime-MVA boost groups than either the MVA or Adenoviral alone groups. Although cell-mediated immunity is crucial in protection against TB, the role of antibody responses in protection against TB is of increasing interest; sera from TB contacts with high tuberculin-specific IgG titers has been shown to block proliferation of PBMC cultured with tuberculin [18]. Furthermore, it was demonstrated that human mycobacteria-specific IgG enhanced complement activation by *M*.*tb* [19].

We have shown that an AERAS-402 prime-MVA85A boost regimen increases durability of antigen specific T-cell responses, increases the frequency and polyfunctionality of CD8+ T-cells and increases antibody responses when compared with AERAS-402 or MVA85A vaccination alone. This improved cellular and humoral immunogenicity may be important in protection against TB. One of the challenges faced by the TB vaccine field is the low immunogenicity induced by candidate vaccines in the target population, compared to UK adults. This adenoviral prime-MVA boost strategy may increase the magnitude and durability of vaccine-induced responses and broaden the quality of the immune response in the target population. Further studies with adenoviral prime-MVA85A boost regimens are merited to optimise intervals between vaccinations, dose and route of immunisation, and also to evaluate this strategy in the target population in TB high burden countries.

## Supporting Information

S1 CONSORT Checklist CONSORT Checklist. (DOC)

S1 Protocol Study protocol. (PDF)
