# Supplementary material for: A Phase I, Open-Label Trial, Evaluating the Safety and Immunogenicity of Candidate Tuberculosis Vaccines AERAS-402 and MVA85A, Administered by Prime-Boost Regime in BCG-Vaccinated Healthy Adults
Source: PLoS One. 2015 Nov 3;10(11):e0141687. doi: 10.1371/journal.pone.0141687 (PMC4631471; doi:10.1371/journal.pone.0141687)
Supplement: S1 Protocol — (PDF) [file pone.0141687.s002.pdf]

CLINICAL TRIAL PROTOCOL

Trial reference: TB032

Title: A Phase I, Open Label Trial to Evaluate the Safety and Immunogenicity of AERAS-402 followed by MVA85A in BCG vaccinated adults

Trial Reference:	TB032
Protocol Version Number:	5.0
Date:	2 December 2013
EudraCT number:	2012-002007-18
REC Reference:	12/SC/0283
Sponsor:	University of Oxford
Chief Investigator:	Professor Helen McShane
Local Safety Committee Chair:	Dr Brian Angus
Funding body:	Aeras
Author:	SJ Vermaak, J Meyer

Statement of Compliance

Investigator Agreement

"I have read this protocol and agree to abide by all provisions set forth therein. I agree to comply with the International Conference on Harmonisation Tripartite Guideline on Good Clinical Practice."

------------------------------	------------------------------	------------------------------
Chief Investigator	Investigator Signature	Date

Professor Helen McShane

Confidentiality Statement

This document contains confidential information that must not be disclosed to anyone other than the Sponsor, the Investigator Team, and members of the Independent Ethics Committee. This information cannot be used for any purpose other than the evaluation or conduct of the clinical investigation without the prior written consent of Professor Helen McShane.

Contents

Statement of Compliance 2
Contents 3
Key Roles and General Information 4
List of abbreviations 6
List of protocol amendments 7
1. Trial Summary 11
2. Background Information 14
3. Objectives 24
4. Trial Design 25
5. Selection and Withdrawal of Subjects 26
6. Trial Vaccine, Medicines & Devices 28
7. Trial Schedule 30
8. Adverse Events 32
9. Statistics and Data Handling 36
10. Quality Assurance and Quality control 37
11. Finance 38
12. Ethical and Regulatory Considerations 38
13. Indemnity/Compensation/Insurance 39
14. References 40

KEY ROLES AND GENERAL INFORMATION

Trial Centres:

Centre for Clinical Vaccinology and Tropical Medicine (CCVTM)
University of Oxford
Churchill Hospital
Old Road
Headington
Oxford
OX3 7LE

The Wellcome Trust Clinical Research Facility (WTCRF)
The Queen Elizabeth Hospital
Edgbaston,
Birmingham
B15 2TH

Chief Investigator:

Professor Helen McShane
CCVTM

Principal Investigator:

Prof Paul Moss
WTCRF

Lead Investigator(s):

Dr Joel Meyer
CCVTM

Dr Matt O'Shea
WTCRF

Dr Zita-Rose Manjaly
CCVTM

Dr Sharon Sheehan
CCVTM

Dr Alice Minhinnick
CCVTM

Project Managers:

Dr Alison Lawrie & Mrs Samantha Vermaak
CCVTM

Local Safety Monitor:

Dr Brian Angus
Nuffield Department of Medicine
University of Oxford
Old Road
Headington
Oxford
OX3 7BN

Senior Immunologist:

Dr Iman Satti
The Jenner Institute
University of Oxford
Old Road Campus Research Building
Oxford OX3 7DQ

**Sponsor:**	University of Oxford<br>Clinical Trials and Research Governance Joint Research Office<br>University of Oxford<br>Block 60, Churchill Hospital<br>Old Road<br>Headington<br>Oxford<br>OX3 7LE
**Monitor:**	Appledown Clinical Research Ltd<br>Greenland’s Lane<br>Prestwood<br>Bucks<br>HP16 9QX

List of abbreviations

**AE**	Adverse Event
**ALT**	Alanine Aminotransferase
**BCC**	Basal cell carcinoma
**BCG**	Bacille Calmette-Guérin
**CCVTM**	Centre for Clinical Vaccinology and Tropical Medicine
**CD4**	Cluster of differentiation four (T-cells)
**CD8+**	Cluster of differentiation eight (T-cells)
**CIS**	Carcinoma *in situ*
**CFP10**	Culture filtrate protein 10kDa
**CRF**	Case Report Form
**DNA**	Deoxyribonucleic Acid
**EC**	Ethics Committee
**ELISPOT**	Enzyme-linked Immunospot
**ESAT-6**	Early-secreted antigenic target 6kDa protein
**FDA**	Food and Drug Administration
**GCP**	Good Clinical Practice
**GMO**	Genetically Modified Organism
**GP**	General Practitioner (Family Doctor)
**HBsAg**	Hepatitis B Surface Antigen
**HBV**	Hepatitis B Virus
**HCG**	Human Chorionic Gonadotrophin
**HCV**	Hepatitis C Virus
**HIV**	Human Immunodeficiency Virus
**HLA**	Human Leukocyte Antigen
**ICH**	International Committee on Harmonisation
**ICS**	intracellular cytokine staining
**ID**	Intradermal
**IDT**	IDT Biologika GmbH
**IFN-γ**	Interferon Gamma
**IL-2**	Interleukin-2
**IM**	Intramuscular
**IMP**	Investigational Medicinal Product
**LSC**	Local Safety Committee
M. tb	*Mycobacterium tuberculosis*
**MVA**	Modified vaccinia Virus Ankara
**NHS**	National Health Service
**OETC**	Oxford Emergent Tuberculosis Consortium
**PBS**	Phosphate buffered saline
**SAE**	Serious Adverse Event
**SAR**	Serious Adverse Reaction
**SOP**	Standard Operating Procedure
**SUSAR**	Suspected Unexpected Serious Adverse Event
**TB**	Tuberculosis
**TNF-α**	Tumour necrosis factor-α
**TST**	Tuberculin skin test

List of protocol amendments

Protocol v5.0 Substantial Amendment

Section	From	Changed To
Key roles and responsibilities		Addition of new Investigator Alice Minhinnick
1	Trial duration estimated at 18 months	Trial duration estimated at 24 months
1	Follow up duration of 203 days and 140 days	Follow up duration of 365 days
1 - Schedule of procedures	9 visits	10 visits
2.2	*Additional follow up period information*	An additional visit at 12-18 months has been added. This additional visit will allow a further set of bloods to be taken for immunological analysis. Our preliminary trial results have suggested that valuable information will be gained from bloods at this additional timepoint.
13.2 Compensation		Compensation rates updated to take extra visit into account

Protocol v4.0 Substantial Amendment

Section	From	Changed To
1	45 healthy adult subjects	38 healthy adult subjects
1	Group A: 15 subjects receiving 2 doses of AERAS-402 followed by 1 dose of MVA85A	Group A: 12 subjects receiving 2 doses of AERAS-402 followed by 1 dose of MVA85A
	Group B: 15 subjects receiving 1 dose of AERAS-402 followed by 1 dose of MVA85A	Group B: 14 subjects receiving 1 dose of AERAS-402 followed by 1 dose of MVA85A
	Group C: 15 subjects receiving 3 doses of AERAS-402	Group C: 12 subjects receiving 3 doses of AERAS-402
2.2	There will be 15 participants in each of the three trial groups.	There will be 12 participants in group A, 14 participants in group B and 12 participants in group C.
4.2	Table 5. Trial groups<br>Second column: Sample size<br>15 Group A,15 Group B, 15 Group C	Table 5. Trial groups<br>Second column: Sample size<br>12 Group A,14 Group B, 12 Group C
9.1	Fifteen subjects will be recruited into each of the three arms of the trial.	Twelve subjects will be recruited to group A, 14 subjects to group B and 12 subjects to group C.

Protocol v3.0 Substantial Amendment

Section	From	Changed To
	Sponsor address: OX3 7LJ	OX3 7LE
7.1	*Additional recruitment method*	Direct mail-out: this will involve obtaining names and addresses of adults via the most recent Electoral Roll. The contact details of individuals who have indicated that they do not wish to receive postal mail-shots would be removed prior to the investigators being given this information. The company providing this service is registered under the Data Protection Act 1998. Investigators would not be given dates of birth or ages of individuals but the list supplied would only contain names of those aged between 18-55 years (as per the inclusion criteria).
7.1	*Additional recruitment method*	In addition we will be contacting volunteers from two previous trials TB024 (REC ref 10/H0605/22; group A only) and TB030 (REC ref MSD/IDREC/C1/2012/7) to invite them to participate in this trial TB032. Wording of this letter (which will be sent by email or to their last known address) will be submitted with the application for ethical approval.

Protocol v2.0 Substantial Amendment

Section	From	Changed To
		Additional site and staff
List of abbrev.		Tidied up
1	Start date changed from 1st July 2012	10th September 2012
1		Vital signs added to screening visit in schedule of trial procedures
4.2		All enrolment and all vaccinations will take place at the Oxford University CCVTM.
5.1	Resident in or near Oxford for the duration of the trial period	Resident in or near Oxford (for CCVTM) or Birmingham (for WTCRF; and able to travel to Oxford for vaccinations) for the duration of the trial period
5.2	• Inability to discontinue daily medications other than the following during the trial: oral contraceptives, vitamins, nonprescription nutritional supplements, aspirin, antihistamines, antihypertensives, antidepressants, inhaled steroids and bronchodilators.	• Inability to discontinue daily medications other than the following during the trial: oral contraceptives, vitamins, nonprescription nutritional supplements, aspirin, antihistamines, antihypertensives, antidepressants, inhaled steroids, bronchodilators, and any other stable, regular medication not deemed to have an impact on safety or immunogenicity.
7.3	Subjects will be enrolled on the vaccination visit (day 0).	Subjects will be enrolled on the vaccination visit (day 0) at the Oxford University CCVTM.
8.8		Any SAE occurring in WTCRF volunteers will be notified to the Chief Investigator by the Principal Investigator within 1 working day of the Principal Investigator becoming aware of the SAE.

8.8	If the SAE is a SUSAR it will be reported to the EC, MHRA, and FDA (the latter in the case of AERAS-402 only) within 7 days of the Chief Investigator having first knowledge of the event for fatal and life-threatening cases and within 15 days for all other SUSARs (and the report copied to the Sponsor).	If the SAE is a SUSAR it will be reported to the EC, MHRA, **Aeras**, and the FDA (the latter in the case of AERAS-402 only) within 7 days of the Chief Investigator having first knowledge of the event for fatal and life-threatening cases and within 15 days for all other SUSARs (and the report copied to the Sponsor).
8.8	The DSUR for AERAS-402 will be written by Aeras and submitted by Aeras/University of Oxford.	The DSUR for AERAS-402 will be written and submitted by Aeras/University of Oxford.
9.2	The Chief Investigator will be responsible for collecting, recording, analysing, and storing all the data accruing from the trial. These tasks may be delegated to other Investigators. Data will be stored on paper CRFs held in a key-locked cabinet at the CCVTM. Some data may be duplicated anonymously into an electronic Microsoft Excel™ file on the CCVTM secure server.	The Chief Investigator will be responsible for collecting, recording, analysing, and storing all the data accruing from the trial. These tasks may be delegated to other Investigators. Data will be stored on paper CRFs held in a key-locked cabinet at the CCVTM and on the OpenClinica™ database (which is stored electronically on a secure encrypted University of Oxford server), Both clinical sites will use OpenClinica, however Oxford is responsible for the data. Some data may be duplicated anonymously into an electronic Microsoft Excel™ file on the CCVTM secure server.
11	This trial will be financed by Aeras.	This trial will be financed by Aeras and OETC.
13.2	Subjects will be compensated *pro rata* for their time and travel and for trial procedures while participating in the trial, amounting to a total of £325 for Group B and £375 for Groups A and C. If extra visits are required, compensation will be adjusted accordingly.	Subjects will be compensated *pro rata* for their time and travel and for trial procedures while participating in the trial, amounting to a total of £325 for Group B and £375 for Groups A and C in Oxford, and £365 for Group B and £435 for Groups A and C in Birmingham (due to longer travelling times for vaccination visits). If extra visits are required, compensation will be adjusted accordingly.
Throughout		Minor typographical corrections

Protocol v1.2 Non-substantial Amendment

Section	From	Changed To
1		Removal of HLA test from the screening visit to the day of vaccination (test only required as a baseline exploratory reading)
1		Removal of urinalysis test from D0 visit as test only required at screening
1		Following the exploratory immunology plan, small changes were made to the blood volume taken at some visits (overall volume across trial remained the same)
2.3		Correction of typographical error for blood volume total across trial to 429mL
7.2		Samples may be shipped to Aeras and/or other parties involved in this trial in anonymised form for immunological analysis.

8.8	If the SAE is a SUSAR it will be reported to the EC, and MHRA within 7 days of the Chief Investigator having first knowledge of the event for fatal and life-threatening cases and within 15 days for all other SUSARs… SAEs which are not SUSARS will not normally be reported to the EC and MHRA unless there is a clinically important increase in occurrence rate, an unexpected outcome, or a new event that is likely to affect safety of trial subjects	If the SAE is a SUSAR it will be reported to the EC, MHRA **and FDA (the latter in the case of AERAS-402 only)** within 7 days of the Chief Investigator having first knowledge of the event for fatal and life-threatening cases and within 15 days for all other SUSARs… SAEs which are not SUSARs will not normally be reported to the EC, MHRA,**or FDA** unless there is a clinically important increase in occurrence rate, an unexpected outcome, or a new event that is likely to affect safety of trial subjects.

1. Trial Summary

**Trial Title**	A Phase I, Open Label Trial to Evaluate the Safety and Immunogenicity of AERAS-402 followed by MVA85A in BCG vaccinated adults
**Trial Identifier**	TB032
**Clinical Phase**	I
**Active Ingredients of Vaccines**	AERAS-402, MVA85A
**Finished Products**	AERAS-402, MVA85A
**Dose(s)**	1 x 10^{11} vp AERAS-402; 1 x 10^{8} pfu MVA85A
**Route(s)**	Intramuscular (AERAS-402) or intradermal (MVA85A) needle injection in the deltoid region of the arm
**Trial Interventions**	Intramuscular or intradermal vaccination<br>Venepuncture
**Principal Investigator**	Professor Helen McShane
**Trial Centres**	Centre for Clinical Vaccinology and Tropical Medicine (CCVTM)<br>Churchill Hospital, Old Road, Headington<br>Oxford, OX3 7LE<br><br>The Wellcome Trust Clinical Research Facility (WTCRF)<br>The Queen Elizabeth Hospital, Edgbaston,<br>Birmingham B15 2TH
**Planned Trial Period**	Prospective start date for enrolment 10th September 2012
**Trial Duration**	Estimated at 24 months
**Primary Objectives**	To evaluate the safety profile of multiple doses of AERAS-402 alone, compared to one and two doses of AERAS-402 followed by MVA85A in healthy, BCG vaccinated adults
**Secondary Objectives**	To evaluate and compare the immune responses as described by flow cytometric intracellular cytokine staining, antibody serology, and Elispot assays of multiple doses of AERAS-402 alone, compared to one and two doses of AERAS-402 followed by MVA85A in healthy, BCG vaccinated adults
**Planned Sample Size**	38 healthy adult subjects<br>Group A: 12 subjects receiving 2 doses of AERAS-402 followed by 1 dose of MVA85A<br>Group B: 14 subjects receiving 1 dose of AERAS-402 followed by 1 dose of MVA85A<br>Group C: 12 subjects receiving 3 doses of AERAS-402
**Allocation Method**	Enrolment of groups A and B in parallel will proceed first, and subjects will be enrolled into group C once groups A and B are complete.
**Vaccination Schedule**	(see schedule below)
**Follow-up Duration**	365 days from day 0 (Groups A and C)<br>365 days from day 0 (Group B)
**Blood Sampling Schedule**	See schedule of trial procedures below
**Primary Evaluation Criteria**	Actively and passively collected data on adverse events
**Secondary Evaluation Criteria**	Laboratory markers of cell mediated immunity in blood

Vaccination Schedule:

	Dose 1	Dose 2	Dose 3
Group A	AERAS-402 (day 0)	AERAS-402 (day 28)	MVA85A (day 119)
Group B	AERAS-402 (day 0)	MVA85A (day 56)	Not applicable
Group C	AERAS-402 (day 0)	AERAS-402 (day 28)	AERAS-402 (day 119)

Schedule of trial procedures - Groups A and C

**Attendance number**	**1**	**2**	**3**	**4**	**5**	**6**	**7**	**8**	**9**	**10**
**Timeline (days)***	S	0	28	42	56	119	126	147	203	365-548
**Timeline (weeks)***	S	0	4	6	8	17	18	21	29	52-78
**Time windows (days)**			±5	±5	±5	±7	±2	±5	±14	
**Inclusion/exclusion criteria**	X		X			X				
**Review contra-indications**	X	X								
**Informed consent**	X									
**Medical history**	X	(X)	(X)	(X)	(X)	(X)	(X)	(X)	(X)	(X)
**Physical examination**	X									
**Height and weight**	X									
**Vital signs**	X	X	X	X	X	X	X	X	X	X
**Urinalysis**	X									
**β-HCG urine test**	X	X	X			X				
**Vaccinations**		X	X			X				
**Local & systemic events/reactions**		X	X	X	X	X	X	X	X	X
**Diary cards provided**		X	X			X				
**Diary cards collected**			X	X			X			
**Site of injection examination**		X	X	X	X	X	X	X	X	X
**Biochemistry (4mL)**	X		X	X		X	X			
**Haematology (2mL)**	X		X	X		X	X			
**HBV, HCV, HIV (5mL)**	X									
**HLA profile (4mL)**		X								
**Exploratory immunology incl ELISPOT and ICS (10-55mL)****	X	X	X	X	X	X	X	X	X	X
**Blood vol (mL)**	**21**	**59**	**51**	**51**	**45**	**61**	**51**	**45**	**45**	**45**
**Cumulative blood vol (mL)**	**21**	**80**	**131**	**182**	**227**	**288**	**339**	**384**	**429**	**474**

S Screening
X Event scheduled to occur
(X) If considered necessary, emphasising any complaint or change in medications
* Timeline is approximate only, as exact timings (± time windows) of visits relate to the actual (not intended) date of the previous visit
** Exploratory immunology at screening only 10mls, 45-55mls at all other time points.
Grey Highlights vaccination day

Table 1. Schedule of trial procedures for Groups A and C

Schedule of trial procedures - Group B

**Attendance number**	**1**	**2**	**3**	**4**	**5**	**6**	**7**	**8**	**9**
**Timeline (days)***	S	0	14	28	56	63	84	140	365 - 548
**Timeline (weeks)***	S	0	2	4	8	9	12	20	52-78
**Time windows (days)**			±3	±5	±5	±2	±5	±14	
**Inclusion/exclusion criteria**	X	X			X				
**Review contra-indications**	X	X			X				
**Informed consent**	X								
**Medical history**	X	(X)	(X)	(X)	(X)	(X)	(X)	(X)	(X)
**Physical examination**	X								
**Height and weight**	X								
**Vital signs**	X	X	X	X	X	X	X	X	X
**Urinalysis**	X								
**β-HCG urine test**	X	X			X				
**Vaccinations**		X			X				
**Local & systemic events/reactions**		X	X	X	X	X	X	X	X
**Diary cards provided**		X			X				
**Diary cards collected**			X			X			
**Site of injection examination**		X	X	X	X	X	X	X	X
**Biochemistry (4mL)**	X		X		X	X			
**Haematology (2mL)**	X		X		X	X			
**HBV, HCV, HIV (5mL)**	X								
**HLA profile (4mL)**		X							
**Exploratory immunology incl ELISPOT and ICS (10-55mL)****	X	X	X	X	X	X	X	X	X
**Blood vol (mL)**	**21**	**59**	**51**	**45**	**61**	**51**	**45**	**45**	**45**
**Cumulative blood vol (mL)**	**21**	**80**	**131**	**176**	**237**	**288**	**333**	**378**	**423**

S	Screening
X	Event scheduled to occur
(X)	If considered necessary, emphasising any complaint or change in medications
*	Timeline is approximate only, as exact timings (± time windows) of visits relate to the actual (not intended) date of the previous visit
**	Exploratory immunology 10mls at screening, 45-55mls at all other time points.
Grey	Highlights vaccination day

Table 2. Schedule of trial procedures for Group B

2.1. Background Information

Introduction

Mycobacterium tuberculosis (*M. tb*) is a pathogen with worldwide preponderance which infects humans causing tuberculosis (TB), a transmissible disease resulting in very high mortality and morbidity. A third of the world's population is latently infected with *M. tb*, these people carry a 10% lifetime risk of developing active life-threatening disease[1]. In 2007, there were 9.27 million new cases worldwide and 1.77 million people died of TB[2]. Co-infection with human immunodeficiency virus (HIV) greatly increases risk of TB reactivation and death[3, 4]. Diagnosis is challenging and drug treatment can be prolonged, harmful, costly and complex. For these reasons an effective vaccine is a global public health priority.

The Bacille Calmette-Guérin (BCG) vaccine is the only licensed TB vaccine and it has been administered globally to several billion people over a 70 year period[5]. Although it does not protect against pulmonary TB in many parts of the world, it is effective in preventing disseminated TB disease including tuberculous meningitis in childhood[4, 6, 7]. Recently heterologous "prime-boost" vaccination strategies, in which two different candidate vaccines expressing antigens in common are given weeks or months apart, have generated strong and sustained cellular immune responses correlating with a TB protective effect in preclinical animal models. In such a "prime-boost" strategy, BCG would therefore be an ideal priming vaccine.

MVA85A is a recombinant attenuated Modified Vaccinia virus Ankara expressing the *M. tb* antigen 85A. This antigen is highly conserved in all strains of BCG and *M. tb*, and is immunodominant in both animal and human studies. Developed by the University of Oxford, MVA85A has now been administered to over 2100 human individuals with an excellent safety profile. It has been shown to be highly immunogenic as a booster vaccine in BCG-primed subjects[8], and a large phase IIb efficacy trial in BCG-vaccinated infants in South Africa is currently under way.

AERAS-402 is a live recombinant serotype 35 replication deficient adenovirus vector expressing a fusion protein (TB-S) of three *M. tb* antigens: 85A, 85B and TB10.4. Developed by Crucell Holland B.V., a biotechnology company in the Netherlands, AERAS-402 has now been administered to over 350 human individuals with an acceptable safety profile. It has been shown to be immunogenic in adults and infants, and a large phase IIb efficacy trial in BCG-vaccinated infants in Africa is underway.

Description and pre-clinical experience of MVA and MVA85A

Modified vaccinia virus Ankara (MVA) is a highly attenuated strain of vaccinia virus which cannot replicate in human cells. It is known to be highly immunogenic and is therefore suitable for use as a viral vector in new vaccine development. It has an excellent safety record as it was administered intradermally to approximately 120,000 people during the smallpox eradication campaign[9-12], and has since been used in numerous clinical trials of candidate vaccines against viral, mycobacterial and protozoal infections[13, 14]. Meanwhile recombinant MVA vaccines administered by respiratory mucosal and gut mucosal routes have demonstrated protective efficacy and elicited strong immune responses in both rodents and non-human primates[15].

Antigen 85A is a highly conserved antigen expressed by *M. tb*, BCG, and all other mycobacterial species sequenced to date. It is a 32-kDa protein, and is an enzyme, mycolyl transferase, which is involved in cell wall biosynthesis[16]. Antigen 85A is highly immunodominant in both animal and human studies[17-19], and protects against *M. tb* challenge in mice, guinea pigs and non-human primates[20, 21]. The recombinant MVA85A vaccine incorporates the 1176 base pair gene for antigen 85A into the viral DNA allowing expression of this secreted antigen.

Experiments in mice, guinea pigs, cattle, and non-human primates have shown that a prime-boost schedule of vaccination with BCG followed by MVA85A, either intradermally, intramuscularly or mucosally, can improve protective efficacy against subsequent *M. tb* challenge, compared to BCG alone [21-24]. Animal toxicity studies using the intradermal route of administration revealed no differences from PBS-injected controls apart from irritation at the site of administration.

Clinical studies with MVA85A

Over 2100 subjects have now received MVA85A, of which 12 were by the intramuscular route and the remainder intradermally. 16 phase I/II clinical studies of MVA85A have been completed, a further 3 are ongoing. These are summarised in table 3.

Protocol	Phase	Population	Treatment groups[a]	N	Trial Status
TB002	Phase I open label non-randomised	Healthy BCG-naïve adults, UK	5×10^7 pfu MVA85A (Days 0 & 21)	14	Completed
GM920	Phase I open label non-randomised	Healthy BCG-naïve or<br>BCG-vaccinated adults, Gambia	5×10^7 pfu MVA85A (Days 0 & 21) BCG naïve<br>5×10^7 pfu MVA85A (Day 0) BCG-vaccinated	11<br>10	Completed
TB004	Phase I open label non-randomised	Healthy BCG-naïve<br>adults, UK	1×10^6 pfu BCG prime (Day 0)<br>5×10^7 pfu MVA85A boost (after 1 month)	10	Completed
TB005	Phase I open label non-randomised	Healthy BCG-vaccinated adults, UK	5×10^7 pfu MVA85A (Day 0)	21	Completed
TB007	Phase I open label non-randomised	Healthy adults latently infected with *M. tb*,<br>UK	5×10^7 pfu MVA85A (Day 0)	12	Completed
TB008	Phase I open label non-randomised	Healthy BCG-naïve or vaccinated adults &<br>adolescents, South Africa	5×10^7 pfu MVA85A (Day 0) adults<br>5×10^7 pfu MVA85A (Day 0) adolescents	24<br>12	Completed
TB009	Phase I open label non-randomised	Healthy BCG-vaccinated adults, UK	1×10^7 pfu MVA85A (Day 0)<br>1×10^8 pfu MVA85A (Day 0)	12<br>12	Completed
TB010	Phase I open label non-randomised	HIV-positive adults, UK	5×10^7 pfu MVA85A (Day 0)<br>1×10^8 pfu MVA85A (Day 0)	10<br>10	Completed
TB011	Phase I open label non-randomised	Adults infected with *M. tb*, HIV, or both,<br>South Africa	5×10^7 pfu MVA85A (Day 0) TB<br>5×10^7 pfu MVA85A (Day 0) HIV<br>5×10^7 pfu MVA85A (Day 0) TB & HIV<br>5×10^7 pfu MVA85A (Day 0) HIV on ARV treatment	12<br>12<br>12<br>12	Completed
TB012	Phase II open label non-randomised	Healthy BCG-vaccinated children & infants,<br>South Africa	<u>Stage 1</u><br>EPI alone (Day 0)<br>2.5×10^7 pfu MVA85A with EPI (Day 0)<br>2.5×10^7 pfu MVA85A alone (Day 0)[b]<br>EPI alone (Day 0)<br>5×10^7 pfu MVA85A with EPI (Day 0)<br>5×10^7 pfu MVA85A alone (Day 0)[b]<br><u>Stage 2</u><br>EPI alone<br>5×10^7 pfu MVA85A with EPI (Day 0)<br>5×10^7 pfu MVA85A alone (Day 0)[b]	 12<br>12<br>12<br>12<br>12<br>12<br><br>47<br>47<br>48	<u>Stage 1</u><br>Completed<br><br><br><br><br><br><u>Stage 2</u><br>Completed
TB014	Phase II open label non-randomised	Healthy BCG-vaccinated<br>children & infants, South Africa	5×10^7 pfu MVA85A (Day 0) children<br>2.5×10^7 pfu MVA85A (Day 0) infants<br>5×10^7 pfu MVA85A (Day 0) infants<br>1×10^8 pfu MVA85A (Day 0) infants<br>Prevenar (variable dose) (Day 0) infants	24<br>36<br>36<br>36<br>36	Completed
TB017	Phase I open label non-randomised	Healthy BCG-vaccinated<br>adults, UK	5×10^7 pfu FP85A (Day 0)<br>5×10^7 pfu MVA85A (Day 0) then 5×10^7 pfu FP85A (Day 28)<br>5×10^7 pfu FP85A (Day 0) then 5×10^7 pfu MVA85A (Day 28)	12<br>12<br>7	Completed
TB018	Phase I open label non-randomised	Healthy BCG-vaccinated adults, UK	1×10^8 pfu MVA85A (Day 0)<br>1×10^8 pfu MVA85A (Day 0) then1 g/kg deuterium labelled glucose (Day 4)<br>1×10^8 pfu MVA85A (Day 0) then1 g/kg deuterium labelled glucose (Day 10)	4<br>4<br>4	Completed
TB019	Phase I open label non-randomised	HIV-infected adults, Senegal	1×10^8 pfu MVA85A (Day 0 & 168)<br>1×10^8 pfu MVA85A (Day 0 & 168) on ARV treatment	12<br>12	Completed
TB020	Phase II double blinded randomised	Healthy BCG-vaccinated HIV-negative<br>infants, South Africa	1×10^8 pfu MVA85A (Day 0)<br>Placebo	1392<br>1392	In follow up
TB021	Phase II double blinded randomised	Healthy HIV-infected adults, South Africa &<br>Senegal	1×10^8 pfu MVA85A (Day 0)<br>Placebo	700<br>700	Enrolling

[a]Intradermal route of administration unless otherwise stated [b]EPI deferred for 1 week

Protocol	Phase	Population	Treatment groups[a]	N	Trial Status
TB022	Phase I open-label randomised	Healthy BCG-vaccinated adults, UK	Intramuscular 1 x 10^8 pfu MVA85A (Day 0)<br>Intradermal 1 x 10^8 pfu MVA85A (Day 0)	12<br>12	Completed
TB023	Phase I open-label non-randomised	Healthy BCG-naïve or vaccinated adults, UK	1x10^8 pfu MVA85A (Day 0) then BCG challenge (Day 28)<br>BCG: 100 µl ~ 2-8 x 10^5 pfu	24<br>24	Completed
TB026	Phase I randomised blinded	Healthy BCG-vaccinated adults, UK	1x10^7 pfu aerosol inhaled MVA85A with intradermal saline placebo<br>1x10^7pfu intradermal MVA85A injection with aerosol inhaled saline placebo	12<br>12	Enrolling

Table 3. Summary of clinical trials of MVA85A

Safety profile of MVA85A
The typical local reaction as a result of intradermal injection is temporary pain, redness and swelling at the site of the injection with a 5-10 mm central red swollen area and a paler red area ranging from 5-10 mm in diameter which peaks at 48 hours post vaccination. At seven days post vaccination, only the central area of redness remains. This fades over a few weeks and is not usually apparent eight weeks after vaccination. The theoretical risk of a Koch reaction, whereby individuals with pre-existing mycobacterial immunity may develop an exaggerated immunopathological response to a boosting immunization, has not become manifest in trials thus far, despite vaccination of 48 latently infected subjects.

Systemic reactions to the vaccine are short-lived, and resemble influenza symptoms including fatigue, headache, malaise, feverishness, and muscle aches. These systemic symptoms are typically mild and occur in 50-90% of all subjects.

There have been 2 possibly related SAEs in our ongoing efficacy trials. Both trials are still blinded and it is not known if the subjects received vaccine or placebo. The first SAE was a hospitalization due to a fever of 39.0^{o}C in an infant, which resolved within 48 hours and no other cause was found. This was deemed an expected reaction after vaccination. The second SAE was in our ongoing trial in HIV-infected adults. This SAE (preferred term: meningitis tuberculous) was judged to be unexpected and possibly related to study vaccine, and is therefore a suspected unexpected serious adverse reaction [SUSAR]).DSMB review did not request unblinding and recommended trial continue.

Dosing
Dose studies of MVA85A have been performed in a step-wise fashion to minimise the risk of a Koch reaction and the incidence of adverse events. The main dose-finding trial compared boosting doses of 1×10^7 pfu and 1×10^8 pfu to previous trials using boosting doses of 5×10^7 pfu. An intradermal dose of 1×10^8 pfu was found to be significantly more immunogenic than the lower doses without concomitant worsening of adverse event profile, and has subsequently been adopted as the standard injectable dose in all subsequent trials.

Immunogenicity
When evaluated by various assays of cellular immunology including interferon gamma (IFN-γ) ELISPOT and intracellular cytokine staining assays, MVA85A has induced a strong and sustained cell-mediated immune response which is hoped to correlate with a TB-protective effect[8]. There are currently two phase IIb efficacy trials of MVA85A, one in BCG-vaccinated infants in South Africa, and a second in HIV infected adults in South Africa and Senegal.

Description and pre-clinical experience of AERAS-402
AERAS-402 is a live recombinant serotype 35 replication deficient adenovirus vector expressing a fusion protein (TB-S) of three *M. tb* antigens: 85A, 85B and TB10.4. Antigens 85A and 85B are both part of the secreted mycolyl transferase complex involved in mycobacterial cell wall maintenance. Antigen TB10.4 is a member of the secreted ESAT-6 family of proteins that are virulence factors mediating the entry of mycobacteria into cells. The TB-S components are all immunogenic, eliciting T cell responses. Ag85A, Ag85B and members of the ESAT-6 family have also shown activity in protection of animals from *M. tb* challenge. The vaccine DNA causes the production of the vaccine fusion protein by host cells and this fusion protein is immunogenic, producing the biological effect of the vaccine.

AERAS-402 given alone by the intramuscular route is immunogenic in mice and mini-pigs (swine) and exhibits a boost phenomenon when mice have been pre-inoculated (primed) with BCG. AERAS-402 given alone by the intramuscular route exhibits protective activity in the mouse *M. tb* challenge model. Attempts to demonstrate an augmentation of protection above that induced by BCG in the guinea pig *M. tb* challenge model have yielded a nonsignificant statistical trend toward prolonged survival. With the exception of some mild injection site histological findings, AERAS-402 given intramuscularly or intravenously does not exhibit any toxicity in guinea pigs or in mini-pigs (swine) either during life or in autopsies when given in multiple doses with a total dose higher than that planned for humans.

Clinical studies with AERAS-402

A total of 13 clinical studies (10 Phase I, 3 Phase II) of AERAS-402 have been completed or are ongoing, in which a total of 276 adult subjects have been vaccinated to date. Of the adult subjects that have been vaccinated, 170 have received AERAS-402, 45 have received placebo, and 12 have received BCG alone; treatment assignment remains blinded for the remaining adult subjects. A total of 391 infant subjects have been vaccinated to date, of which 196 have received AERAS-402 and 67 have received control; treatment assignment remains blinded for the remaining infant subjects. The doses of AERAS-402 in these studies range from 1.5 x 10^8 to 1.0 x 10^{11} vp. These are summarized in Table 4.

Protocol	Phase	Population	Treatment groups	N	Trial Status
C-001-402	Phase I, open-label, non-randomized	Healthy adults, BCG-naïve, US	• 3 x 10^8 vp AERAS-402 (Day 0)<br>• 3 x 10^9 vp AERAS-402 (Day 0)<br>• 3 x 10^{10} vp AERAS-402 (Day 0)<br>• 3 x 10^{10} vp AERAS-402 (Days 0, 56)	8<br>8<br>8<br>8	Completed
C-003-402	Phase I, double-blind, randomized	Healthy adults, BCG-vaccinated, South Africa	• 3 x 10^8 vp AERAS-402 (Day 0)<br>• 3 x 10^9 vp AERAS-402 (Day 0)<br>• 3 x 10^{10} vp AERAS-402 (Day 0)<br>• 3 x 10^{10} vp AERAS-402 (Days 0, 56)<br>• Placebo (Day 0 or Days 0, 56)	7<br>7<br>7<br>8<br>11	Completed
C-004-402	Phase I, double-blind, randomized	Healthy adults, BCG-vaccinated, India	• 3 x 10^{10} vp AERAS-402 (Days 0, 28)<br>• Placebo (Days 0, 28)	8<br>4	Completed
C-008-402	Phase I, double-blind, randomized	Healthy adults, BCG-naïve, US	• BCG prime (Day 0), 3 x 10^{10} vp AERAS-402 (Days 84, 112)<br>• BCG prime (Day 0), placebo (Days 84, 112)	8<br>9	Completed
C-009-402	Phase I, double-blind, randomized	Healthy adults, BCG-naïve, US	• BCG prime (Day 0), 3 x 10^{10} vp AERAS-402 (Days 168, 196)<br>• BCG prime (Day 0), placebo (Days 168, 196)<br>• BCG prime (Day 0), BCG booster (Day 168) (open-label)	8<br>9<br>5	Completed
C-010-402	Phase II, double-blind, randomized	Adults recently treated for pulmonary TB, South Africa	• 3 x 10^8 vp AERAS-402 (Day 0)<br>• 3 x 10^9 vp AERAS-402 (Day 0)<br>• 3 x 10^{10} vp AERAS-402 (Days 0, 42)<br>• Placebo (Day 0 or Days 0, 42)<br><br>[Stratified by time from start of TB treatment (between 1 and 4 months prior to Study Day 0; at least 12 months prior to Study Day 0)]	10<br>20<br>31<br>11	Completed
C-012-402	Phase I, double-blind, randomized,	Healthy adults BCG-vaccinated, QFT-G(+) or QFT-G(-), without evidence of TB, Kenya	• 3 x 10^{10} vp AERAS-402 (Days 0, 56)<br>• Placebo (Days 0, 56)<br><br>[Stratified by QFT-G(+) or QFT-G(-)]	16<br>4	Completed
C-017-402	Phase II, double-blind, randomized	Adults infected with HIV, BCG-vaccinated, South Africa	• 3 x 10^{10} vp AERAS-402 (Days 0, 28)<br>• 1 x 10^{11} vp AERAS-402 (Days 0, 28)<br>• Placebo (Days 0, 28)	13<br>387-587<br>400-600	Enrolling (n=26)
C-018-402	Phase I, double-blind, randomized	Healthy infants, BCG-vaccinated, South Africa	• 1.5 x 10^8 vp AERAS-402 (Day 0)<br>• 1.5 x 10^9 vp AERAS-402 (Day 0)<br>• 1.5 x 10^{10} vp AERAS-402 (Day 0)<br>• 1.0 x 10^{11} vp AERAS-402 (Days 0, 56)<br>• Prevnar or 0.9% sodium chloride (Day 0 or Days 0, 56)	10<br>10<br>10<br>12<br>15	Completed
C-021-402	Phase I, double-blind, randomized	Healthy adults, BCG-naïve, US	• BCG prime (Day -84), 3 x 10^{10} vp AERAS-402 (Days 0, 28)<br>• BCG prime (Day -84), placebo (Days 0, 28)	10<br>3	Completed [11 of 13 planned subjects were enrolled]
C-022-402	Phase I, double-blind, randomized	Healthy adults, BCG-naïve, US	• BCG prime (Day 0), 3 x 10^{10} vp AERAS-402 (Days 84, 112)<br>• BCG prime (Day 0), 1 x 10^{11} vp AERAS-402 (Days 84, 112)<br>• BCG prime (Day 0), placebo (Days 84, 112)	8<br>12<br>4	Completed

Protocol	Phase	Population	Treatment groups	N	Trial Status
C-029-402	Phase II, double-blind, randomized	Healthy infants, BCG-vaccinated, various Africa countries	Dose-finding phase<br>• 1.5 x 10^{10} vp AERAS-402 (Days 0, 28)<br>• 3.0 x 10^{10} vp AERAS-402 (Days 0, 28)<br>• 1.0 x 10^{11} vp AERAS-402 (Days 0, 28)<br>• Placebo (Days 0, 28)<br><br>Safety and efficacy phase<br>• AERAS-402 (Days 0, 28)<br>• Placebo (Days 0, 28)<br><br>[dose of AERAS-402 in safety and efficacy phase to be selected based on safety data from dose-finding phase]	 51<br>51<br>52<br>52<br><br><br>1100-2000<br>1100-2000	 Follow-up<br><br><br><br><br><br>Enrolling (n=261)
C-031-402	Phase I, double-blind, randomized	Healthy adults, received 2 doses of AERAS-402 in C-022-402, US	• 3 x 10^{10} vp AERAS-402 (Day 0)<br>• 1 x 10^{11} vp AERAS-402 (Day 0)<br>• Placebo (Day 0)	1<br>2<br>2	Completed

Table 4. Summary of clinical trials of AERAS-402

Safety profile of AERAS-402
Clinical experience with AERAS-402 in both BCG vaccinated and unvaccinated individuals so far reveals no related serious adverse events in studies which have been unblinded. In an ongoing blinded large phase IIb efficacy trial in BCG-vaccinated infants in Africa, 1 serious adverse event (tachypnea) judged to be unexpected and possibly related to blinded study vaccine has been reported (this event is therefore a SUSAR). The DMC conducted an unblinded review of the SUSAR and other non-serious tachypnea events. There was an even distribution of tachypnea events between placebo and AERAS-402 with no safety signal noted, and continuation of dosing and enrolment in the study was permitted by the DMC. In adults, AERAS-402 provokes mild to moderate local injection site reactions with some cases of severe injection site pain. It appears that there may be a dose-dependent increase in the frequency and severity of injection site reactions. Moderate to severe constitutional symptoms such as fever, headache, malaise, myalgia, sore throat, arthralgia, and fatigue have been attributed to AERAS-402. There appears to be an increased incidence of mild to moderate fever and myalgia in adult subjects receiving the highest dose level of AERAS-402 (1×10^{11} vp) compared to those receiving lower dose levels of AERAS-402. Mild to moderate upper respiratory and gastrointestinal manifestations have been attributed to AERAS-402. Mild abnormalities of partial thromboplastin time, prothrombin time, and hepatic enzymes, and mild to moderate abnormalities of leukocyte parameters with some severe decreases in neutrophil count, have been attributed to AERAS-402.

Adverse events attributed to AERAS-402 in infants include mild to moderate local injection site reactions, and constitutional symptoms (most commonly malaise, rhinitis, body temperature increased (fever), and fatigue). A dose-dependent increase in the frequency of injection site pain, malaise, and fever was observed in infants. Some changes in laboratory parameters were also attributed to AERAS-402, the most common (occurred in 2 or more subjects) being hemoglobin decreased, ALT increased, and neutrophil count decreased. Most of the laboratory changes considered related to AERAS-402 were mild, although some subjects had moderate hemoglobin decreased and 1 subject had transient severe neutrophil count decreased.

Dosing
Dose studies of AERAS-402 have been performed in a step-wise fashion to minimise the incidence of adverse events. Doses have ranged from 1×10^8 to 1×10^{11} vp, all of which have demonstrated an acceptable safety profile. In the dose-finding phase of the ongoing phase IIb trial in infants, the 1×10^{11} vp dose level showed an immune responder rate by multifunctional CD8+ T cell ICS of 30-50%. This dose level is now being used in the efficacy phase of this ongoing phase IIb trial.

Immunogenicity
When evaluated by the intracellular cytokine staining (ICS) assay, AERAS-402 has induced CD8+ responses in adults and infants after 1 or 2 doses, with a boosting effect observed after the second dose in some subjects.

2.2. Rationale

Hypothesis

AERAS-402 presents *Mycobacterium tuberculosis* antigens in the setting of a new, live, replication-deficient adenovirus vaccine that may increase T-cell-mediated immunity and thus protection from tuberculosis. AERAS-402 appears immunogenic in adults and infants with an acceptable safety profile but primarily stimulates a CD8 T cell response. MVA85A also presents tuberculosis antigens in the setting of a live but non-replicating pox virus vaccine to increase T-cell immunity, and thus protection against tuberculosis, and primarily stimulates a CD4 T-cell response. Additionally, preclinical and clinical studies in HIV and malaria respectively have shown that adenovirus prime followed by MVA boost is effective at inducing high levels of immunity and protection[25, 26]. This trial will evaluate the safety and immunogenicity of MVA85A administered to BCG vaccinated adults who have received one or two vaccinations with AERAS-402. The trial's rationale is to evaluate the additive effect of MVA85A administered to recipients of two regimens of AERAS-402.

Trial design

TB032 will be a Phase I, open-label, non-randomised trial with three trial arms.

Group numbers

There will be 12 participants in group A, 14 participants in group B and 12 participants in group C.

Vaccine dosage

Trials of MVA85A to date have established 1 x 10^8 pfu as the optimal dose for intradermal injection in adults. The dose of AERAS-402 is based on already performed adult and infant studies that indicate a dose of 1 x 10^{11} vp to be immunogenic with an acceptable safety profile.

Trial population

The overall investigational approach with our MVA85A trials is to develop an effective prime-boost vaccination strategy to prevent TB infection, with BCG as the priming vaccine. As BCG is protective in childhood, it is routinely given at birth in TB endemic countries but is also in widespread use in children and adolescents in much of the rest of the world. Therefore, as in previous trials of intradermal MVA85A, we propose to vaccinate BCG-primed subjects. This is the first trial in which MVA85A will be used in combination with AERAS-402, therefore the trial will be conducted in a healthy adult population.

Time points

Prior BCG – trial enrolment time window

The specified period for prior receipt of BCG is such that no less than six months must have elapsed between prior BCG vaccination and subsequent trial enrolment. This is to allow for the peak BCG-induced immune response to have occurred, which is usually around 2-4 weeks post BCG vaccination.

Screening – trial enrolment time window

Enrolment should take place no longer than 90 days following the date of screening appointment. If more than 21 days elapse, the screening visit should be repeated in full prior to enrolment in order to minimise the risk to participants of any new unidentified health problems having arisen during that period.

Follow up period

The follow up period will be 12-18 months from first vaccination in accordance with findings from previous trials in which adequate safety data and reliable markers of immunogenicity have been obtained in this time interval. The final visit will allow immunological analysis of longer-term durability of the vaccines.

2.3. Risks and Benefits

Potential risks

The potential risks to participants in this trial include risks associated with:

1. Venepuncture

Localised bruising and discomfort can occur at the site of venepuncture. Infrequently fainting may occur. The total volume of blood drawn over a 7 month period will be 429 mL which should not compromise the health of these healthy individuals.

2. Vaccination

MVA85A and AERAS-402 have undergone thorough pre-clinical testing. MVA85A has now been administered to over 2100 healthy human individuals and AERAS-402 has been administered to over 350 human individuals with a good safety profile demonstrated for both vaccines. The potential known adverse events associated with vaccination are:

Local reaction from intradermal vaccination (MVA85A)

The typical local reaction as a result of intradermal injection is temporary pain, redness and swelling at the site of the injection with a 5-10 mm central red swollen area and a paler red area ranging from 5-10 mm in diameter which peaks at 48 hours post vaccination. At seven days post vaccination, only the central area of redness remains. This fades over a few weeks and is not usually apparent eight weeks after vaccination. Scaling occurs as part of the healing process, and itching is possible.

Local reaction from intramuscular vaccination (AERAS-402)

The typical local reaction as a result of intramuscular injection is temporary pain, heat, redness and swelling at the site of the injection.

Systemic reactions

Constitutional influenza-like symptoms such as fatigue, headache, malaise, feverishness, and muscle aches can occur with any vaccination lasting for 2-3 days. All of these adverse events have been reported with both MVA85A and AERAS-402.

Allergic reactions

Allergic reactions from mild to severe may occur in response to any constituent of a vaccine preparation. Anaphylaxis is extremely rare but can occur in response to any vaccine. No anaphylactic reactions have been seen to either experimental vaccines used in this trial.

Known potential benefits

Subjects are not expected to benefit directly from participation in this trial but may obtain additional protection against TB infection as a result of vaccination with the candidate vaccines AERAS-402 and MVA85A. Subjects will also gain some information about their general health as a result of the screening history, examination, blood tests and urine tests. It is hoped that their contribution will enable the development of a safe and successful vaccine for TB.

3. OBJECTIVES

3.1. Primary Objectives

To evaluate the safety profile of multiple doses of AERAS-402 alone, compared to one and two doses of AERAS-402 followed by MVA85A in healthy, BCG vaccinated adults.

3.2. Secondary Objectives

To evaluate and compare the immune responses as described by flow cytometric intracellular cytokine staining and Elispot assays of multiple doses of AERAS-402 alone, compared to one and two doses of AERAS-402 followed by MVA85A in healthy, BCG vaccinated adults.

4.1. Description and Justification of Trial Design

This is a phase I trial to evaluate the safety profile of multiple doses of AERAS-402 alone, compared to one and two doses of AERAS-402 followed by MVA85A in healthy, BCG vaccinated adults.

4.2. Trial groups

Enrolment of groups A and B in parallel will proceed first, and subjects will be enrolled into group C once groups A and B are complete. All enrolment and all vaccinations will take place at the Oxford University CCVTM.

Arm of trial	Sample size	Intervention
Group A	12	Day 0: Intramuscular AERAS-402 1 x 10^{11} vp<br>Day 28: Intramuscular AERAS-402 1 x 10^{11} vp<br>Day 119: Intradermal MVA85A 1 x 10^{8} pfu
Group B	14	Day 0: Intramuscular AERAS-402 1 x 10^{11} vp<br>Day 56: Intradermal MVA85A 1 x 10^{8} pfu
Group C	12	Day 0: Intramuscular AERAS-402 1 x 10^{11} vp<br>Day 28: Intramuscular AERAS-402 1 x 10^{11} vp<br>Day 119: Intramuscular AERAS-402 1 x 10^{11} vp

Table 5. Trial groups

4.3. Endpoints

Primary endpoint

The primary endpoint is safety data in the three groups, as assessed by the frequency, incidence, and nature of AEs and SAEs during the trial. This will be collected in three main ways:

- Information about local and systemic reactions and adverse events will be collected at each visit by medical history and use of diary cards, and site of injection examinations at selected visits.
- Vital signs will be recorded at each visit.
- Safety blood tests (haematology, biochemistry) at days 28, 42, 119, 126 (Groups A and C), and days 14, 56, 63 (Group B).

Secondary endpoints

The secondary endpoint is immunogenicity data in the three groups. This will be obtained from exploratory immunological laboratory investigations on blood samples taken at each visit, and will include the following:

- Elispot assays of IFN-γ secreted CD4+ and CD8+ T cells of subjects stimulated with a pool of mycobacterial peptides.
- Flow cytometry estimation of T cell IFN-γ, TNF-α, and IL-2 production after stimulation with mycobacterial peptides, as quantified by ICS.

4.4. Safety

Safety monitoring

The local safety committee (LSC) will provide safety oversight for this trial. Its chair will be a clinician with extensive and relevant experience of infectious disease and clinical trials work. At the time of writing the chair is Dr Brian Angus, Clinical Tutor in Medicine, Honorary Consultant Physician and Director of the Centre for Tropical Medicine at the University of Oxford. There will be a minimum of two other appropriately qualified committee members as specified in the site-specific SOP for safety reporting.

The chair of the LSC will be contacted for advice and independent review in the following situations:

- Following any SAE deemed to be possibly, probably, or definitely related to the trial vaccine.
- Any other situation where the Investigator feels independent advice or review is important.

Discontinuation of the trial

The trial will be discontinued in the event of any of the following:

- New scientific information is published to indicate that subjects in the trial are being exposed to undue risks as a result of administration of the IMPs, or as a result of the trial procedures or follow-up schedule.
- Serious concerns about the safety of the IMPs arise as a result of one or more vaccine related SAE(s) occurring in the subjects enrolled in this or any other ongoing trial of the MVA85A or AERAS-402 vaccines.
- For any other reason at the discretion of the Investigator.

4.5. Data Collection

Information will be entered into the CRF contemporaneously at each visit. Results of laboratory investigations and immunological data will be entered into the CRF. The laboratory reports, diary cards and CRFs will be the source data for this trial.

5. Selection and Withdrawal of Subjects

5.1. Inclusion Criteria

Subjects must meet all of the following criteria to enter the trial:

- Healthy adult aged 18-55 years
- Resident in or near Oxford (for CCVTM) or Birmingham (for WTCRF; and able to travel to Oxford for vaccinations) for the duration of the trial period
- No relevant findings in medical history or on physical examination
- Confirmation of prior vaccination with BCG not less than 6 months prior to projected trial vaccination date (by visible BCG scar on examination or written documentation)
- Allow the Investigators to discuss the individual's medical history with their GP
- Use effective contraception for the duration of the trial period (females only)
- Refrain from blood donation during the trial
- Give written informed consent
- Allow the Investigator to register subject details with a confidential database to prevent concurrent entry into clinical trials
- Agrees to avoid elective surgery for the duration of the trial

- Has a body mass index (BMI) between 18 and 33 (weight/height^2) by nomogram
- Able and willing (in the Investigator's opinion) to comply with all the trial requirements

5.2. Exclusion Criteria

Subjects must meet none of the following criteria to enter the trial:

- Laboratory evidence at screening of latent *M. tb* infection as indicated by a positive ELISPOT response to ESAT6 or CFP10 antigens[b]
- Clinical, radiological, or laboratory evidence of current active TB disease[a]
- Shared a residence within one year prior to day 0 with an individual on anti-tuberculosis treatment or with culture- or smear-positive pulmonary tuberculosis
- Previous treatment for active or latent tuberculosis infection
- Received a TST within 90 days prior to day 0
- Received a systemic antibiotic within 14 days prior to day 0
- Inability to discontinue daily medications other than the following during the trial: oral contraceptives, vitamins, nonprescription nutritional supplements, aspirin, antihistamines, antihypertensives, antidepressants, inhaled steroids, bronchodilators, and any other stable, regular medication not deemed to have an impact on safety or immunogenicity.
- Previous vaccination with candidate vaccine MVA85A or candidate vaccine FP85A or any other recombinant MVA or adenoviral vaccine; AERAS-402; or any other investigational *M. tb* vaccine
- Clinically significant history of skin disorder, allergy, immunodeficiency (including HIV), autoimmune disease, cancer (except BCC or CIS), cardiovascular disease, gastrointestinal disease, liver disease, renal disease, endocrine disorder, neurological illness, psychiatric disorder, drug or alcohol abuse
- History of serious psychiatric condition
- Concurrent oral or systemic steroid medication or the concurrent use of other immunosuppressive agents
- History of anaphylaxis to vaccination or any allergy likely to be exacerbated by any component of the trial vaccine, including eggs
- Any abnormality of screening blood or urine tests that is deemed to be clinically significant or that may compromise the safety of the subject in the trial[a]
- Positive HBsAg, HCV or HIV antibodies[a]
- Female currently lactating, confirmed pregnancy or intention to become pregnant during trial period
- Use of an investigational medicinal product or non-registered drug, live vaccine, or medical device other than the trial vaccine for 30 days prior to dosing with the trial vaccine, or planned use during the trial period
- Administration of immunoglobulins and/or any blood products within the three months preceding the planned trial vaccination date
- Any other significant disease, disorder, or finding, which, in the opinion of the Investigator, may either put the subject at risk or may influence the result of the trial or may affect the subject's ability to participate in the trial

[a]Subjects who are excluded from the trial because they have been discovered during screening procedures to be suffering from a previously undiagnosed condition thought to require further medical attention will be referred appropriately to their GP or an NHS specialist service for further investigation and treatment.

[b]Subjects discovered to have evidence of latent *M. tb* infection as defined by a positive ELISPOT test will be referred for a plain chest x ray and reviewed with the TB nurse specialists and considered for chemoprophylaxis. If there is any evidence of active TB disease either on clinical or radiological grounds, further investigation and treatment will be offered under the supervision of a consultant physician in respiratory or infectious diseases.

5.3. Withdrawal Criteria

Subjects may be withdrawn from the trial early:

- By withdrawing voluntarily
- On the decision of the Investigator
- On the advice of the LSC

The Investigator may withdraw the subject for any of the following reasons:

- Confirmed pregnancy during the trial
- Any adverse event which results in inability to comply with trial procedures
- Ineligibility either arising during the trial or retrospectively (having been overlooked at screening)
- Significant protocol deviation
- Subject non-compliance with trial requirements
- Loss to follow up (applies to a subject who does not return for protocol trial visits, is not reachable by telephone or other means of communication and/or is not able to be located)

The reason for withdrawal will be recorded in the CRF. Subjects withdrawn from the trial may be replaced on the decision of the Investigator. If the subject is withdrawn due to an AE, the Investigator will arrange for appropriate specialist management or follow up visits or telephone calls until the AE has resolved or stabilised. The regulatory authorities will be informed in a timely manner. The extent of follow up after premature discontinuation will be determined by the Investigator but will be at least for the whole trial period, and if pregnant, until pregnancy outcome.

5.4. Vaccination postponement criteria

Vaccination will not proceed on the scheduled day in any of the following situations:

- The subject has a temperature > 37.5^{o}C
- The Investigator judges the subject to have an acute moderate or severe illness (whether febrile or not)
- The subject has received a live non-trial vaccine within the preceding 30 days
- The subject is taking antibiotics
- The Investigator has any other concern that vaccination may not be in the subject's best interests

In this case the subject may be vaccinated at a later date or withdrawn from the trial at the discretion of the Investigator.

6. Trial Vaccine, Medicines & Devices

6.1. Candidate TB vaccine MVA85A

Description

The MVA85A vaccine consists of the attenuated vaccinia virus MVA vector with a 1176 base-pair insert, which is almost the complete *M. tb* gene for Ag85A, with the tissue plasminogen activator (TPA) signal sequence preceding the N terminus and a monoclonal antibody tag (pk) at the C terminus. Expression of the antigen 85A DNA sequence is regulated by the vaccinia P7.5 early/late

promoter. MVA85A is manufactured under Good Manufacturing Practice conditions by IDT Biologika GmbH (IDT), Germany.

Formulation

MVA85A is supplied as a liquid in glass vials. Each vial contains 400 µL of vaccine at a concentration of 8.8 x 10^8 pfu/mL in 10mM Tris buffer. The dose of MVA85A to be used in this trial will be 1 x 10^8 pfu (114 µL) in a single intradermal injection into the deltoid region of the upper arm.

Vaccine supply and product storage

MVA85A will be shipped from IDT directly to the Clinical Biomanufacturing Facility (CBF). The vaccine will be batch certified and labelled for release by a qualified person (QP) at the University of Oxford. The vaccine will be stored at -80°C in a secure, temperature-monitored freezer at the CCVTM, University of Oxford, Churchill Hospital.

Dispensing and administration

All movements of the trial vaccines will be documented. Vaccine accountability, storage, shipment and handling will be in accordance with local SOPs and other relevant local forms. On vaccination day, vaccines will be allowed to thaw to room temperature and will be administered within 1 hour. The vaccine will be administered intradermally over the deltoid region of the upper arm, according to the site-specific SOP. Subjects will stay in the unit for 60 minutes (±10 minutes) after vaccination. During the administration of the vaccine, monitoring equipment, oxygen, medicines including bronchodilators and resuscitation equipment will be immediately available for the management of anaphylaxis and bronchospasm.

In order to minimise dissemination of the recombinant vectored vaccine virus into the environment, a number of measures will be instituted during and following vaccination:

- The intradermal/intramuscular injection site will be covered with a dressing after vaccination to absorb any virus that may leak out through the needle track. The dressing will be removed from the injection site after 30 minutes.
- All disposable items including needles, vials, dressings, and protective clothing will be disposed of as GMO waste by autoclaving, in accordance with the current approved SOPs and standard UK practice.

6.2. Candidate TB vaccine AERAS-402

Description

AERAS-402 is a live recombinant adenoviral vaccine containing a replication deficient serotype 35 adenovirus (Ad35) derived adenovector, which harbors a transgene encoding a *M. tb* fusion protein. This fusion protein, TB-S, combines three independent antigenic domains: Ag85A, Ag85B and TB10.4. AERAS-402 is manufactured under Good Manufacturing Practice conditions by Crucell Holland B.V. in the Netherlands.

Formulation

AERAS-402 is supplied as a frozen liquid in single use clear glass vials. Each vial contains an extractable 0.5 mL of vaccine at a concentration of 2 x 10^{11} vp/mL in 10mM Tris buffer. The dose of AERAS-402 to be used in this trial will be 1 x 10^{11} vp (0.5 mL) in a single intramuscular injection into the deltoid region of the upper arm.

Vaccine Supply and product storage

AERAS-402 will be shipped from Fisher Clinical Services (Allentown, PA, USA) directly to the Clinical Biomanufacturing Facility (CBF). The vaccine will be batch certified and labelled for release by a

qualified person (QP) at the University of Oxford. The vaccine will be stored at -80°C in a secure, temperature-monitored freezer at the University of Oxford, Churchill Hospital.

Dispensing and administration

All movements of the trial vaccine will be documented. Vaccine accountability, storage, shipment and handling will be in accordance with local SOPs and other relevant local forms. On vaccination day, vaccines will be allowed to thaw to room temperature and will be administered within 1 hour of thawing. The vaccine will be administered intramuscularly over the deltoid region of the upper arm, according to the site-specific SOP. Subjects will stay in the unit for 60 minutes (±10 minutes) after vaccination. During the administration of the vaccine, monitoring equipment, oxygen, medicines including bronchodilators and resuscitation equipment will be immediately available for the management of anaphylaxis and bronchospasm.

7. Trial Schedule

7.1. Recruitment

Subjects may be recruited by use of an advertisement formally approved by the ethics committee and distributed or posted in the following places:

- In public places (including NHS hospitals and university buildings) with the agreement of the owner or proprietor
- In newspapers or other literature for circulation
- On a website operated by our group or with the agreement of the owner or operator
- By e-mail distribution to a group or list only with the express agreement of the network administrator or with equivalent authorisation
- On stalls or stands at exhibitions or fairs
- Direct mail-out: this will involve obtaining names and addresses of adults via the most recent Electoral Roll. The contact details of individuals who have indicated that they do not wish to receive postal mail-shots would be removed prior to the investigators being given this information. The company providing this service is registered under the Data Protection Act 1998. Investigators would not be given dates of birth or ages of individuals but the list supplied would only contain names of those aged between 18-55 years (as per the inclusion criteria).

A copy of intended advertising will be submitted with the initial application for ethical approval and any significant changes to this advert will be submitted as an amendment to the ethics committee for approval before use.

In addition we will be contacting volunteers from two previous trials TB024 (REC ref 10/H0605/22; group A only) and TB030 (REC ref MSD/IDREC/C1/2012/7) to invite them to participate in this trial TB032. Wording of this letter (which will be sent by email or to their last known address) will be submitted with the application for ethical approval.

7.2. Screening

Subjects will be offered a screening visit if they express an interest in participating in the trial. They will be assigned a subject identifier number and provided with and asked to read the subject information sheet at least 24 hours prior to attending for screening. At the screening visit the general process of screening will be outlined and all questions about the screening process and the trial answered. The following general principles will be emphasised:

- Participation in the trial is entirely voluntary
- Refusal to participate involves no penalty or loss of medical benefits
- The subject may withdraw from the trial at any time
- The subject is free to ask questions at any time to allow him or her to understand the purpose of the trial and the procedures involved
- The trial involves administration of an unlicensed vaccine(s)
- There is no direct benefit from participating
- The subject's GP will be contacted to corroborate their medical history if possible

The aims of the trial and all tests to be carried out will be explained by the Investigator or the Clinical Research Nurse. The subject will be given the opportunity to ask about details of the trial, and will then have time to consider whether or not to participate. If they do decide to participate, they will sign and personally date two copies of the consent form, one for them to take away and keep, and one for the Investigator. These forms will also be signed and dated by the Investigator or Clinical Research Nurse.

Having agreed to undergo screening, a baseline medical history (including concomitant medication) and physical examination will be performed. Inclusion and exclusion criteria will be checked using a tabulated format. Demographic and occupational data relating to risk of prior TB exposure will be collected. Vital signs will be checked. Subjects will be counselled by one of the Investigators for HIV, Hepatitis B and Hepatitis C testing. Blood tests as specified in the schedule of procedures will be taken. Urinalysis and a pregnancy test will be performed. The total duration of the screening visit will be approximately one and a half hours.

Subjects will be informed that there may be leftover samples of their blood (after all testing for this trial is completed), and that such samples may be stored up to a maximum of 15 years for possible future research. Samples may be shipped to Aeras and/or other parties involved in this trial in anonymised form for immunological analysis. Subjects will be able to decide if they will permit such future use of any leftover samples. If they elect not to permit this, all of those leftover samples will be discarded after the required period of storage to meet Good Clinical Practice (GCP) and regulatory requirements.

The subject's general practitioner will be contacted with the written permission of the subject after satisfactory screening as notification that the subject is taking part in the trial and to ascertain any significant medical history.

7.3. Enrolment

Subjects who fulfil the screening criteria, satisfy all the inclusion criteria, and meet none of the exclusion criteria will be enrolled into the trial and their visits scheduled in the calendar. The ongoing eligibility of the subject will be reviewed on the day of enrolment and any new events, medications, or changes to the screening documents recorded. Subjects will be enrolled on the vaccination visit (day 0) at the Oxford University CCVTM. The vaccination visit will last approximately two hours, which includes a 60 minute follow up period in the clinic for observation after vaccination. All pre-vaccination and follow up visits will last approximately 15 to 30 minutes.

Enrolment should take place no longer than 90 days following the date of screening appointment. If more than 90 days elapse, the screening visit should be repeated in full prior to enrolment.

7.4. Trial Termination

Every reasonable effort will be made to maintain protocol compliance and participation in the trial. If a subject is prematurely withdrawn from the trial for any reason (detailed in section 5.3), the reason for early trial withdrawal will be recorded in the CRF. Reasons for discontinuation of the trial are detailed in section 4.4. The trial will be completed when the last subject enrolled into the trial has completed their final follow up visit.

8. Adverse Events

An adverse event (AE) is a general term encompassing any untoward medical occurrence occurring in any phase of the clinical trial in a clinical trial subject, whether or not considered causally related to a trial intervention. This includes local and systemic symptoms and signs occurring in the trial period, exacerbations (but not day-to-day fluctuations) of pre-existing conditions, intercurrent illnesses, abnormal laboratory results, or dosing errors.

All AEs can be classified in terms of their severity (absent, mild, moderate, severe), causality (no relationship, unlikely, possible, probable, definite), expectedness (unexpected or expected), and seriousness (not serious or serious).

8.1. Clinical Assessment

All AEs (whether reported by the subject or solicited by the Investigator) will be clinically assessed at each visit and recorded with start and stop dates and details of any treatment undergone.

This information will be **collected** as follows:

- Subjects are asked to complete diary cards for 7 days after AERAS-402 or MVA85A vaccination. They are provided with a thermometer and tape measure to enable daily recording of temperature and local redness and swelling at the injection site.
- Outside the diary card periods, expected related local and systemic AEs (listed in table 6 below) will be solicited, in other words specifically asked about at each visit, and graded by severity and causality (as detailed in sections 8.2 and 8.3).
- Subjects will be given the opportunity to report any other new symptoms since the last visit with start and stop dates and any treatments undergone.
- Vital signs will be performed in the normal manner at each visit.

	**Adverse event**
**Local**	Pain at the injection site
	Redness at the injection site
	Swelling at injection site
	Warmth at the injection site
	Itch at the injection site
	Scaling at the injection site
	Axillary lymphadenopathy
**Systemic**	Documented fever (oral temperature >= 38.0° C)
	Symptoms of feverishness
	Malaise
	Arthralgia
	Headache
	Myalgia
	Nausea / vomiting
	Fatigue
	Diarrhoea
	Dysuria
	Conjunctivitis
	Sore throat
	Upper respiratory tract infection

Table 6: Routinely solicited local and systemic adverse events

Diary card AEs, reported AEs, and vital signs will be recorded in the subject's CRF at each visit.

8.2. Severity Assessment

AEs will be graded by severity as follows (where 0 = absent, 1 = mild, 2 = moderate, 3 = severe):

**Adverse event**	**Grade**	**Measurement**
Tenderness or pain at injection site	0	No pain at all
	1	Painful on touch; easily tolerated
	2	Painful when limb is moved; interferes with daily activity
	3	Severe pain at rest; prevents daily activity
Redness at injection site	0	0 mm
	1	1 – 50 mm
	2	51 – 100 mm
	3	> 100 mm
Swelling at injection site	0	0 mm
	1	1 – 20 mm
	2	21 – 50 mm
	3	> 50 mm
Fever (oral)	0	<38.0^{o}C
	1	38.0 – 38.4 ^{o}C
	2	38.5 – 38.9 ^{o}C
	3	>39.0 ^{o}C
Other AEs	0	Absence of symptom
	1	Awareness of symptom but tolerated; transient or mild discomfort; little or no medical intervention required
	2	Discomfort enough to cause limitation of usual activity; some medical intervention or therapy required
	3	Incapacitating, absent from work, or bed rest required; hospitalisation possible

Table 7. Severity grading for AEs

8.3. Causality Assessment

The following guidelines will be used to assess the relationship of all AEs to the administration of the vaccine, with the premise that all local reactions will be considered causally related to the vaccination:

0 = No relationship:
- No temporal relationship to vaccine; <u>and</u>
- Alternate aetiology (clinical state, environmental or other interventions); <u>and</u>
- Does not follow known pattern of response to the vaccine

1 = Unlikely relationship:
- Unlikely temporal relationship to vaccine; <u>and</u>
- Alternate aetiology likely (clinical state, environmental or other interventions); <u>and</u>
- Does not follow known typical or plausible pattern of response to the vaccine

2 = Possible relationship:
- Reasonable temporal relationship to vaccine; <u>or</u>
- Event not readily produced by clinical state, environmental or other interventions; <u>or</u>
- Similar pattern of response to that seen with the vaccine

3 = Probable relationship:
- Reasonable temporal relationship to vaccine; <u>and</u>
- Event not readily produced by clinical state, environment, or other interventions <u>or</u>

- Known pattern of response seen with the vaccine

4 = Definite relationship:

- Reasonable temporal relationship to vaccine; _and_
- Event not readily produced by clinical state, environment, or other interventions; _and_
- Known pattern of response seen with the vaccine

The Investigator and the Local Safety Monitor both determine causality. It is expected that communication and consultation may occur in the assessment of the causality of AEs. The greatest degree of causal relationship (definite > probable > possible > unlikely related > not related) determined by either the Investigator or Local Safety Monitor after their discussions will determine the ultimate classification of the AE. Definite (4), probable (3) and possible (2) are considered to be related. No relationship (0) and unlikely (1) are considered to be unrelated.

8.4. Assessment of Seriousness

All AEs are assessed for severity (mild, moderate, severe) as described above and expectedness. In addition, the *seriousness* of an AE relates to its outcome.

Serious Adverse Events (SAEs)

A serious adverse event (SAE) is any AE that results in one of the following outcomes, whether or not considered related to the vaccine:

- Death
- Life-threatening event (this refers to an event in which, in the view of the Investigator, the subject was at immediate risk of death at the time of the event, *not* to an event which hypothetically might have caused death if it were more severe).
- Admission to hospital regardless of length of stay, even if as a precautionary measure for observation, or prolongation of existing hospitalisation. This does not include hospitalisation for a pre-existing condition that has not worsened unexpectedly.
- Persistent or significant disability or incapacity.
- Congenital anomaly or birth defect.
- An important medical event that may, based upon expert medical judgement, jeopardise the subject and/or require intervention to prevent one of the above outcomes. Examples include allergic reactions, blood dyscrasias, or convulsions that do not result in hospitalisation.

8.5. Assessment of Expectedness

SAEs will be assessed for relatedness. SARs must be assessed for expectedness. Expected events are those consistent with the information about the investigational vaccine provided in the Investigator’s Brochure. All other SARs would be classified as unexpected. In this trial there are no expected SARs therefore any SAR would be classified as a SUSAR until such time as a causal relationship to the vaccine can be excluded.

Suspected unexpected serious adverse reactions (SUSARs)

A SUSAR is an SAE that is both unexpected and thought to be related to an investigational product or trial intervention.

8.6. Laboratory Assessments

All laboratory results will be reviewed and recorded in the CRF after being signed by the Investigator. A note will be made of whether each result is normal, abnormal but not clinically significant, or

abnormal *and* clinically significant. In the latter case trial eligibility will be reviewed and necessary referrals or tests will be offered.

8.7. Follow up of Adverse Events

Adverse events will be recorded in the CRF. Those adverse events likely to be related to the vaccine, whether serious or not, which persist at the end of the trial will be followed up by the Investigator until their complete disappearance.

The outcome of any non-serious adverse event occurring within 30 days post-vaccination or any SAE reported during the entire trial will be assessed as:

- Recovered/resolved
- Not recovered/not resolved
- Recovering/resolving
- Recovered with sequelae/resolved with sequelae
- Fatal

Subjects who have moderate or severe ongoing AEs at the completion of the trial will be advised to consult their General Practitioner (National Health Service) if the event is not considered to be related to the vaccine. A follow-up visit will be arranged to manage the problem and to determine the severity and duration of the event, if it is considered to be related to the vaccine. If appropriate, specialist review within the NHS will be arranged.

8.8. Reporting of Serious Adverse Events

All SAEs will be documented accurately and notification deadlines respected as specified in the site-specific SOP. SAEs will be reported immediately by the Investigator to the Sponsor or person with appropriate responsibility delegated from the Sponsor (Chief Investigator), and to the Local Safety Committee (see section 4.4). This will be performed by emailing an electronic version of the completed SAE Initial Report Form (VC004F1). Any relevant information concerning the SAE that becomes available after the SAE Initial Report Form has been sent (outcome, precise history, results of investigations, copy of hospital report, etc) will be forwarded in a timely manner using the SAE Update Report Form (VC004F2). The Sponsor, or person with appropriate responsibility delegated from the Sponsor (Chief Investigator), is responsible for commencing, maintaining and completing the SAE Sponsor Report Form (VC004F3). The anonymity of subjects shall be respected when forwarding this information.

Any SAE occurring in WTCRF volunteers will be notified to the Chief Investigator by the Principal Investigator within 1 working day of the Principal Investigator becoming aware of the SAE.

If the SAE is a SUSAR it will be reported to the EC, MHRA, Aeras, and the FDA (the latter in the case of AERAS-402 only) within 7 days of the Chief Investigator having first knowledge of the event for fatal and life-threatening cases and within 15 days for all other SUSARs (and the report copied to the Sponsor). Any deaths occurring during the trial will be reported to the Sponsor. For all deaths, available autopsy reports will be made available for reporting to the regulatory authorities.

SAEs which are not SUSARs will not normally be reported to the EC, MHRA,or FDA unless there is a clinically important increase in occurrence rate, an unexpected outcome, or a new event that is likely to affect safety of trial subjects.

In addition to these reporting requirements, the Investigator shall submit once a year throughout the trial a Development Safety Update Report (DSUR) to the EC and MHRA (and a copy to the Sponsor). The DSUR for MVA85A will be written and submitted by University of Oxford. The DSUR for AERAS-402 will be written and submitted by Aeras/University of Oxford.

8.9. Pregnancy

Subjects who become pregnant during the trial after the vaccination may continue trial procedures, including venepuncture but excluding vaccination, if appropriate, at the discretion of the Investigator. Subjects who become pregnant before AERAS-402 or MVA85A vaccination will be withdrawn and will not be vaccinated. The Investigator will collect pregnancy information on any subject who becomes pregnant while participating in this trial. The subject will be followed up to determine the outcome of the pregnancy.

9. Statistics and Data Handling

9.1. Statistics

This is primarily a safety trial with descriptive endpoints. Twelve subjects will be recruited to group A, 14 subjects to group B and 12 subjects to group C. Our previous experience suggests that this sample size is a feasible number to recruit, screen, enrol, and follow up in practical terms, whilst also allowing the determination of any substantial differences in the outcome measures between the three groups, including the frequency of AEs and SAEs and the size of the immune responses generated. The sample size has not been determined with the aim of achieving statistical significance. This sample size is appropriate for a proof-of-concept phase I safety trial.

It is anticipated that the immunological endpoints will not follow a normal distribution. A log transformation will be applied and if the resulting data are normally distributed they will be summarised by group using means and standard deviations. The post-vaccination responses will be compared between the three groups using a t-test. If the resulting data are not normally distributed then medians and interquartile ranges will be used to summarise the data and the Mann Whitney U test will be used for statistical comparisons. An area-under-curve analysis will be used to compare the overall responses over time between the three groups. This analysis will make use of data collected at all time points.

Differences between means or medians between the three groups will be presented along with their 95% confidence intervals. Within each group, paired data will be analysed using either a paired t-test or the Wilcoxon matched pairs test depending on the normality of the data.

9.2. Data handling

The Chief Investigator will be responsible for collecting, recording, analysing, and storing all the data accruing from the trial. These tasks may be delegated to other Investigators. Data will be stored on paper CRFs held in a key-locked cabinet at the CCVTM and on the OpenClinica™ database (which is stored electronically on a secure encrypted University of Oxford server), Both clinical sites will use OpenClinica, however Oxford is responsible for the data. Some data may be duplicated anonymously into an electronic Microsoft Excel™ file on the CCVTM secure server.

Access to the source data documents will be provided if necessary to the EC and MHRA and to the Sponsor for trial-related monitoring and audit. All information relating to the trial and its subjects will be held in strict confidence, and in accordance with ICH E6 GCP and institutional requirements.

Trial records will be held by the Investigator for as long as required by legislation (currently until at least 2 years after the last marketing authorisation for the product or 2 years after discontinuation of

clinical development of an investigational product), initially in a key-locked cabinet at the CCVTM and subsequently in a secure archive facility, and in accordance with the Data Protection Act. Subjects will be assigned individual unique trial numbers for identification on all trial records, except where the use of identifiable information is unavoidable (including on GP correspondence, registration documents, and consent forms).

10. Quality Assurance and Quality control

10.1. Quality Assurance

Investigator procedures

Approved site-specific SOPs will be used at all clinical and laboratory sites.

Modification to protocol

No amendments to this protocol will be made without consultation with, and agreement of, the Sponsor. Any amendments to the trial that appear necessary during the course of the trial must be discussed by the Investigator and Sponsor concurrently. If agreement is reached concerning the need for an amendment, it will be produced in writing by the Chief Investigator and will be made a formal part of the protocol following ethical and regulatory approval.

An administrative change to the protocol is one that modifies administrative and logistical aspects of a protocol but does not affect the subjects' safety, the objectives of the trial and its progress. An administrative change does not require EC approval. However, the EC must be notified whenever an administrative change is made.

The Investigator is responsible for ensuring that changes to an approved trial, during the period for which EC approval has already been given, are not initiated without EC review and approval except to eliminate apparent immediate hazards to the subject.

Protocol deviation

All deviations from the protocol will be documented in a protocol deviation form and filed in the trial master file.

10.2. Monitoring

Data will be evaluated for compliance with the protocol and accuracy in relation to source documents. The trial will be conducted in accordance with procedures identified in the protocol. Regular monitoring will be performed according to ICH GCP. According to applicable SOPs, the Monitors will verify that the clinical trial is initiated, conducted and completed, and data are generated, documented and reported in compliance with the protocol, GCP and the applicable regulatory requirements.

11. FINANCE

This trial will be financed by Aeras and OETC.

12. ETHICAL AND REGULATORY CONSIDERATIONS

12.1. Good Clinical Practice

This trial will be conducted in accordance with the principles of the Declaration of Helsinki as agreed by the World Medical Association General Assembly (Washington 2002), ICH Good Clinical Practice (GCP), the requirements of the Medicines for Human Use (Clinical Trial) Regulations 2004, and local regulatory requirements.

12.2. Ethical Review

A copy of the protocol, proposed informed consent form, other written subject information and the proposed advertising material will be submitted to an independent EC for written approval. The Investigators will submit and, where necessary, obtain approval from the EC for all subsequent substantial amendments to the protocol and informed consent document. The Investigators will notify deviations from the protocol or SAEs occurring at the site to the Sponsor and will notify the EC of these in accordance with local procedures.

12.3. Informed Consent

Written informed consent will be obtained at screening as detailed in section 7.2.

12.4. Serious Breaches

The Medicines for Human Use (Clinical Trials) Regulations contain a requirement for the notification of "serious breaches" to the MHRA within 7 days of the Chief Investigator becoming aware of the breach. A serious breach is defined as “A breach of GCP or the trial protocol which is likely to affect to a significant degree:

- the safety or physical or mental integrity of the subjects of the trial
- the scientific value of the trial”

In the event that a serious breach is suspected, the Sponsor will be contacted as soon as possible.

13. Indemnity/Compensation/Insurance

13.1. Indemnity

Negligent Harm
Indemnity and/or compensation for negligent harm arising specifically from an accidental injury for which the University is legally liable as the Research Sponsor will be covered by the University of Oxford.

Non-Negligent Harm
Indemnity and/or compensation for harm arising specifically from an accidental injury, and occurring as a consequence of the Research Subjects' participation in the trial for which the University is the Research Sponsor will be covered by the University of Oxford.

13.2. Compensation

Subjects will be compensated *pro rata* for their time and travel and for trial procedures while participating in the trial, amounting to a total of £360 for Group B and £410 for Groups A and C in Oxford, and £400 for Group B and £470 for Groups A and C in Birmingham (due to longer travelling times for vaccination visits). If extra visits are required, compensation will be adjusted accordingly.

14. References

1. Dye, C., et al., *Consensus statement. Global burden of tuberculosis: estimated incidence, prevalence, and mortality by country. WHO Global Surveillance and Monitoring Project.* JAMA, 1999. **282**(7): p. 677-86.
2. WHO, *Global tuberculosis control - epidemiology, strategy, financing. http://www.who.int/tb/publications/global_report/2009/en/ (accessed 28 July 2010)*. 2009.
3. Corbett, E.L., et al., *Tuberculosis in sub-Saharan Africa: opportunities, challenges, and change in the era of antiretroviral treatment.* Lancet, 2006. **367**(9514): p. 926-37.
4. Colditz, G.A., et al., *Efficacy of BCG vaccine in the prevention of tuberculosis. Meta-analysis of the published literature.* JAMA, 1994. **271**(9): p. 698-702.
5. Fine, P.E., *The BCG story: lessons from the past and implications for the future.* Rev Infect Dis, 1989. **11 Suppl 2**: p. S353-9.
6. Trunz, B.B., P. Fine, and C. Dye, *Effect of BCG vaccination on childhood tuberculous meningitis and miliary tuberculosis worldwide: a meta-analysis and assessment of cost-effectiveness.* Lancet, 2006. **367**(9517): p. 1173-80.
7. Rodrigues, L.C., V.K. Diwan, and J.G. Wheeler, *Protective effect of BCG against tuberculous meningitis and miliary tuberculosis: a meta-analysis.* Int J Epidemiol, 1993. **22**(6): p. 1154-8.
8. McShane, H., et al., *Recombinant modified vaccinia virus Ankara expressing antigen 85A boosts BCG-primed and naturally acquired antimycobacterial immunity in humans.* Nat Med, 2004. **10**(11): p. 1240-4.
9. Stickl, H., et al., *[MVA vaccination against smallpox: clinical tests with an attenuated live vaccinia virus strain (MVA) (author's transl)].* Dtsch Med Wochenschr, 1974. **99**(47): p. 2386-92.
10. Mayr, A., et al., *[The smallpox vaccination strain MVA: marker, genetic structure, experience gained with the parenteral vaccination and behavior in organisms with a debilitated defence mechanism (author's transl)].* Zentralbl Bakteriol B, 1978. **167**(5-6): p. 375-90.
11. Hochstein-Mintzel, V., H.C. Huber, and H. Stickl, *[Virulence and immunogenicity of a modified vaccinia virus (strain MVA) (author's transl)].* Z Immunitatsforsch Exp Klin Immunol, 1972. **144**(2): p. 104-56.
12. Hochstein-Mintzel, V., et al., *[An attenuated strain of vaccinia virus (MVA). Successful intramuscular immunization against vaccinia and variola (author's transl)].* Zentralbl Bakteriol Orig A, 1975. **230**(3): p. 283-97.
13. Goonetilleke, N., et al., *Induction of multifunctional human immunodeficiency virus type 1 (HIV-1)-specific T cells capable of proliferation in healthy subjects by using a prime-boost regimen of DNA- and modified vaccinia virus Ankara-vectored vaccines expressing HIV-1 Gag coupled to CD8+ T-cell epitopes.* J Virol, 2006. **80**(10): p. 4717-28.
14. Bejon, P., et al., *Safety profile of the viral vectors of attenuated fowlpox strain FP9 and modified vaccinia virus Ankara recombinant for either of 2 preerythrocytic malaria antigens, ME-TRAP or the circumsporozoite protein, in children and adults in Kenya.* Clin Infect Dis, 2006. **42**(8): p. 1102-10.
15. Gherardi, M.M. and M. Esteban, *Recombinant poxviruses as mucosal vaccine vectors.* J Gen Virol, 2005. **86**(Pt 11): p. 2925-36.
16. Belisle, J.T., et al., *Role of the major antigen of Mycobacterium tuberculosis in cell wall biogenesis.* Science, 1997. **276**(5317): p. 1420-2.
17. Denis, O., et al., *Vaccination with plasmid DNA encoding mycobacterial antigen 85A stimulates a CD4+ and CD8+ T-cell epitopic repertoire broader than that stimulated by Mycobacterium tuberculosis H37Rv infection.* Infect Immun, 1998. **66**(4): p. 1527-33.
18. McShane, H., et al., *Enhanced immunogenicity of CD4(+) t-cell responses and protective efficacy of a DNA-modified vaccinia virus Ankara prime-boost vaccination regimen for murine tuberculosis.* Infect Immun, 2001. **69**(2): p. 681-6.
19. Vordermeier, H.M., et al., *Cellular immune responses induced in cattle by heterologous prime-boost vaccination using recombinant viruses and bacille Calmette-Guerin.* Immunology, 2004. **112**(3): p. 461-70.
20. Huygen, K., et al., *Immunogenicity and protective efficacy of a tuberculosis DNA vaccine.* Nat Med, 1996. **2**(8): p. 893-8.

21. Verreck, F.A., et al., *MVA.85A boosting of BCG and an attenuated, phoP deficient M. tuberculosis vaccine both show protective efficacy against tuberculosis in rhesus macaques.* PLoS One, 2009. **4**(4): p. e5264.
22. Williams, A., et al., *Evaluation of vaccines in the EU TB Vaccine Cluster using a guinea pig aerosol infection model of tuberculosis.* Tuberculosis (Edinb), 2005. **85**(1-2): p. 29-38.
23. Goonetilleke, N.P., et al., *Enhanced immunogenicity and protective efficacy against Mycobacterium tuberculosis of bacille Calmette-Guerin vaccine using mucosal administration and boosting with a recombinant modified vaccinia virus Ankara.* J Immunol, 2003. **171**(3): p. 1602-9.
24. Vordermeier, H.M., et al., *Adjuvants induce distinct immunological phenotypes in a cattle tuberculosis vaccine model.* Clin Vaccine Immunol, 2009.
25. Sheehy, S.H., et al., *Phase Ia clinical evaluation of the safety and immunogenicity of the Plasmodium falciparum blood-stage antigen AMA1 in ChAd63 and MVA vaccine vectors.* PLoS One, 2012. **7**(2): p. e31208.
26. Barouch, D.H., et al., *Vaccine protection against acquisition of neutralization-resistant SIV challenges in rhesus monkeys.* Nature, 2012. **482**(7383): p. 89-93.
